# Urban storm water infiltration systems are not reliable sinks for biocides: evidence from column experiments

**DOI:** 10.1038/s41598-021-86387-9

**Published:** 2021-03-31

**Authors:** Marcus Bork, Jens Lange, Markus Graf-Rosenfellner, Birte Hensen, Oliver Olsson, Thomas Hartung, Elena Fernández-Pascual, Friederike Lang

**Affiliations:** 1 Hydrology, Faculty of Environment and Natural Resources, University of Freiburg, 79098 Freiburg, Germany; 2 Soil Ecology, Faculty of Environment and Natural Resources, University of Freiburg, 79098 Freiburg, Germany; 3 Institute of Sustainable and Environmental Chemistry, Leuphana University Lüneburg, 21335 Lünbeburg, Germany; 4 Environmental Research Institute, University College Cork, Cork, T23 XE10 Ireland

**Keywords:** Environmental sciences, Hydrology, Urban ecology, Solid Earth sciences, Hydrogeology

## Abstract

Groundwater quality in urban catchments is endangered by the input of biocides, such as those used in facade paints to suppress algae and fungal growth and washed off by heavy rainfall. Their retention in storm water infiltration systems (SIS) depends, in addition to their molecular properties, on chemical properties and structure of the integrated soil layer. These soil properties change over time and thus possibly also the relevance of preferential flow paths, e.g. due to ongoing biological activity. To investigate the mobility of biocides in SIS, we analyzed the breakthrough of differently adsorbing tracers (bromide, uranine, sulforhodamine B) and commonly used biocides (diuron, terbutryn, octhilinone) in laboratory column experiments of undisturbed soil cores of SIS, covering ages from 3 to 18 years. Despite similar soil texture and chemical soil properties, retention of tracers and biocides differed distinctly between SIS. Tracer and biocide breakthrough ranged from 54% and 5%, to 96% and 54%, respectively. We related the reduced solute retention to preferential transport in macropores as could be confirmed by brilliant blue staining. Our results suggest an increasing risk of groundwater pollution with increasing number of macropores related to biological activity and the age of SIS.

## Introduction

Urban storm water management concepts such as green infrastructure (GI) have become increasingly implemented worldwide for their numerous environmental benefits^1–4^. One example of GI is local infiltration of urban storm water that, in Germany, is required by the German federal water law^5^ due to benefits for micro-climate^6^ and urban hydrology such as reducing storm water quantity^7, 8^, restoring groundwater levels^9–11^ and relieving sewer systems^12^. However, the direct infiltration of urban storm water could lead to groundwater pollution since storm water may be loaded with nutrients^13^, heavy metals^14, 15^ and organic pollutants such as hydrocarbons^16, 17^ and pesticides^18–20^. Recently, biocides used in facade renders and paints to suppress algae and fungal growth^21^ are receiving increasing attention as they can be washed off from facades by heavy rainfall^22–25^. This biocide wash-off occurs during entire rain events^26^, depends on the conditions of the rainfall and on transport processes within the facade material^27^ and happens even fourteen years after painting of the facade^28^.

Biocide-loaded storm water either infiltrates into the soil around houses^29^ or is directed into SIS such as swales and swale-trench-systems. Together with local infiltration of storm water, the purpose of SIS is to reduce moderate contaminant load^30, 31^. Reviews of^17^ and^32^ showed that this may be the case for hydrocarbons or pesticides. However^28^, recently showed that SIS could be pathways for biocides and their transformation products into groundwater. Consequently, further research is needed on factors that may favor the leaching of organic pollutants in SIS.

Generally, the uppermost layer of swales and swale-trench-systems consists of topsoil material^33^. In these layers, organic pollutants such as biocides may be retained by similar processes as in natural soils: transformation, mineralization, plant uptake and sorption (in the following we will call the uppermost soil filter media of SIS only soil). These processes depend on molecular properties of the solutes, on the vegetation cover and on the physical and chemical soil properties such as pH, organic matter (OM) content, texture and structure^34^, which change with time. Furthermore, the retention can be limited by the formation of preferential flow paths. Evidence regarding the relevance of these different processes is missing so far.

Typically, soil pores are clogged by the entry of suspended solids with storm water^35^ thus reducing water infiltration. Nevertheless, the hydraulic efficiency of SIS has been reported to be maintained even after long-term operation^8, 33, 36^. To the best of our knowledge, no study has systematically addressed this contradiction so far. One explanation could be an increased number of preferential flow paths due to root growth and other biological activity that compensates for soil pore clogging. Yet preferential flow paths induce solute transport^37^ as observed for pesticides in agricultural soils^38, 39^. Thus, we investigated three SIS with regard to the influence of preferential flow paths on the transport of biocides.

We analyzed the depth distributions of physical and chemical soil properties and conducted laboratory-scale percolation experiments using undisturbed soil core samples from three SIS established 3, 10 and 18 years ago (F.3, W.10 and V.18). In these experiments we investigated the breakthrough of three commonly used biocides^29, 40^: diuron (3-(3,4-dichlorophenyl)-1,1-dimethylurea), terbutryn (N2-tert-butyl-N4-ethyl-6-methylsulfanyl-1,3,5-triazine-2,4-diamine), and OIT (2-octyl-1,2-thiazol-3-one). Furthermore, we applied a tracer mix of non-adsorbing bromide ($\hbox {Br}^{-}$) and chloride ($\hbox {Cl}^{-}$), together with the variously adsorbing fluorescent dyes uranine (UR) and sulforhodamine B (SRB) that are often used to investigate preferential flow^41–44^. With this multi-tracer approach and a staining of the soil column using brilliant blue, we aimed to investigate pollutant retention capacity of different SIS.

## Results and discussion

### Soil properties

#### Stone content

**Figure 1:**
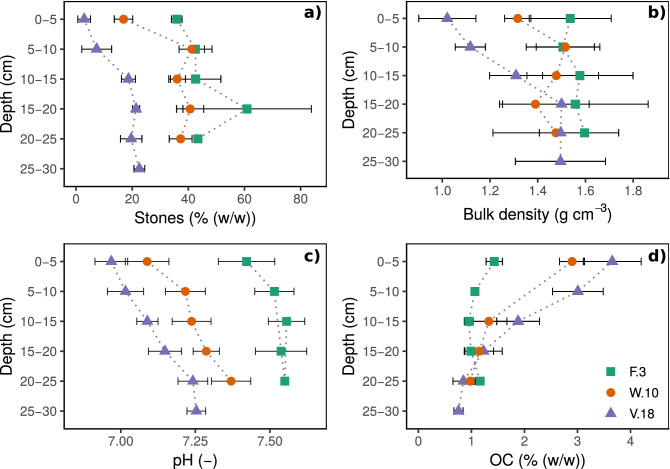
Depth-dependent soil properties: (**a**) stone content [% (w/w)], (**b**) bulk density ($\hbox {g}\,\hbox {cm}^{-3}$), (**c**) pH (0.01 M $\hbox {CaCl}_{2}$) and (**d**) organic carbon content (OC) [% (w/w)] of the three sites F3, W.10, V18. The error bars are the standard deviation ($\hbox {n}=4$).

The stone content ranged from $15\,\pm \,8\%$ (w/w) at V.18 to $44\,\pm \,13\%$ (w/w) at F.3 (Fig. 1a, Table 1). These differences between sites may partly be due to different sources of the raw material used to create the SIS. Further, the stone content increased with depth within the first 15 cm (V.18) and 10 cm (W.10), but remained approximately constant over depth at F.3. Hence, the stone content in the upper layers of the older SIS (W.10 and V.18) was lower than in the lower layers. These depth-related differences at each site may be related to time-dependent developments within the SIS. In the uppermost layers of V.18 and W.10, stone content was comparatively low probably due to input of fine mineral and organic particles by storm water. For the oldest SIS (V.18), this assumption is supported by the field observation of soil material lying on a bricked stone border near the inflow within the SIS.

**Table 1:** Soil properties of SIS.

Site.age	Stones [% (w/w)]	pH^a^ (–)	OC [% (w/w)]	Bulk density ($\hbox {g}\,\hbox {cm}^{-3}$)	Sand	Silt	Clay
					[% (w/w)]		
F.3	$44 \pm 13$	$7.5 \pm 0.1$	$1.1 \pm 0.2$	$1.6 \pm 0.2$	$80 \pm 2$	$16 \pm 2$	$5 \pm 1$
W.10	$34 \pm 10$	$7.2 \pm 0.1$	$1.6 \pm 0.8$	$1.4 \pm 0.2$	$73 \pm 4$	$20 \pm 3$	$7 \pm 1$
V.18	$15 \pm 8$	$7.1 \pm 0.1$	$1.9 \pm 1.2$	$1.3 \pm 0.2$	$57 \pm 7$	$34 \pm 6$	$9 \pm 2$

They are calculated as the mean of all depth steps of four soil cores ± standard deviation. Since the number of depth steps differed between the sites and the number of laboratory repetitions differed, the number of repetitions varied among the methods and the sites (pH: $\hbox {n}=51$–69, OC: $\hbox {n}=17$–23, Stones: $\hbox {n}=16$–22, Bulk density: $\hbox {n}=16$–22; for details see Table S4).

^a^pH in 0.01 M $$\hbox {CaCl}_{2}$$.

#### Bulk density

The bulk density in the upper layers of the different SIS increased in the following order: V.18 < W.10 < F.3 (Fig. 1b, Table 1). At V.18, we observed the strongest change with depth from $1.0\,\pm \,0.1\,\hbox {g}\,\hbox {cm}^{-3}$ (0–5 cm) to $1.5\,\pm \,0.1\,\hbox {g}\,\hbox {cm}^{-3}$ (15–20 cm). In contrast, we observed almost no depth-dependent change of bulk density at the youngest site of F.3 ($1.6\,\pm \,0.2\,\hbox {g}\,\hbox {cm}^{-3}$).

In samples of the older sites of V.18 and W.10, low bulk densities in the uppermost layers compared to deeper layers were probably caused by the activity of macrofauna, an intensive rooting, a higher organic carbon (OC) content and the input of strongly sorted fine material. The older the SIS, the stronger the effect of these factors.

At F.3 the bulk density was relatively high. Here, we supposed an uniform compaction of the soil layer under the topsoil during construction. This assumption was supported by the observation of redox characteristics (iron-red stains next to grey iron-depleted areas) in the soil at approximately 25 cm depth caused by the lack of oxygen due to accumulating water^45^ in compacted soil.

#### Texture

The mean texture of fine soil at all SIS was very similar: 57–80% (w/w) sand, 16–34% (w/w) silt and 5–9% (w/w) clay, since similar textured materials were used for construction to guarantee solute retention and sufficient hydraulic conductivity^31^. Average clay contents of all SIS were within acceptable ranges of Best Management Practice (BMP) claimed by ATV-DVWK A-138 ($<10\%$ (w/w) clay)^31^.

The clay fraction at the oldest SIS (V.18) decreased slightly with depth from 12% (w/w) at 0–5 cm to 7% (w/w) at 20–25 cm (Figure S4) while it remained constant with depth at W.10 and F.3. Since a homogeneous texture after SIS construction can be assumed for each SIS, a higher clay content in the uppermost layers suggested fine particle deposition by storm water, as was observed by^46^.

#### pH

The pH was above 7.0 at nearly all sites and depths (Fig. 1c, Table 1) and was thus within the range of BMP^31^.

The pH increased slightly in the following order: V.18 < W.10 < F.3. These slight pH differences among the SIS could be either due to age (stronger acidification of older soils) or due to different initial pH values of the topsoil materials.

Furthermore, the pH increased with depth at all sites, which was stronger at older sites (V.18 and W.10) than at F.3. These larger depth-gradients at older sites indicate a relationship between pH and age of the sites: older SIS were more acidified in the upper layers than the youngest SIS (F.3) due to the input of acids by precipitation, the production of $\hbox {CO}_{2}$ by soil respiration and the release of protons and organic acids by roots^45^.

#### Organic carbon content

The OC content and the slope of depth-gradients within the upper 15 cm increased from $1.15\,\pm \,0.3\%$ (w/w) at the youngest SIS to $2.85\,\pm \,0.9\%$ (w/w) at the oldest SIS (Fig. 1d, Table 1). This indicates that, in general, OC accumulated during soil development in upper soil layers while the stone content, the bulk density and the pH decreased. Overall, the OC contents of all SIS were within the BMP ranges^31^.

#### Soil development and spatial variability

All soil parameters described above and especially the development of depth gradients indicate continuous soil development (alteration of important soil properties such as pH, texture, OC content and distribution) in the investigated SIS due to physical and chemical processes and sediment input with storm water. Consequently, biological activity also increased, as shown by the observation of macrofauna at V.18 (earthworms) and W.10 (ants) while no macrofauna was observed at F.3. Overall, age of SIS is reflected in the soil properties after only a few years.

We assumed that the spatial variability of the soils within each SIS was relatively small, as probably only one material was used to build up the upper soil layer. As described above, a low spatial variability within each SIS was confirmed by the low variability of analyzed soil properties of the soil samples ($\hbox {n}=4$). Therefore, we assumed in the present study that one soil column with a wide diameter (20 cm) at each SIS can be considered representative of the SIS inflow area. To determine the influence of flow paths on solute transport we thus considered a column experiment without repetition as sufficient to further investigate the differences between the SIS. This is common for such complex column experiments with undisturbed soils (see e.g.^47–49^) since their performance is very time-consuming.

### Percolation experiment

#### Saturated hydraulic conductivity and water flux

The saturated hydraulic conductivity K$_{s}$ ($\hbox {cm}\,\hbox {h}^{-1}$) differed strongly between the soil columns (Table 2). It was lowest in the youngest SIS F.3 ($0.18\,\hbox {cm}\,\hbox {h}^{-1}$), four times higher at W.10 ($0.66\,\hbox {cm}\,\hbox {h}^{-1}$), and approximately six times higher in the oldest SIS V.18 ($0.98\,\hbox {cm}\,\hbox {h}^{-1}$). Water flow velocities at all sites and time steps were in the optimal range for urban swale systems defined by BMP ($0.006\,\hbox {cm}\,\hbox {h}^{-1}< \hbox {q} < 6\,\hbox {cm}\,\hbox {h}^{-1}$)^31^.

**Table 2:** Soil column properties, water fluxes q [average (av), minimum (min) and maximum (max)] and total percolated water (L) during the column experiment in the three soil columns F3, W.10 and V.18.

Column (site.age)	$\hbox {d}_{col}$ (cm)	$\hbox {L}_{col}$ (cm)	$\hbox {PV}_{tot,est}$^a^ (L)	Time (h)	TPS^b^ (L)	$\hbox {q}_{av}$^c^	$\hbox {q}_{min}$^d^	$\hbox {q}_{max}$^c^	$\hbox {K}_{s}$^e^
						($$\hbox {cm}\,\hbox {h}^{-1}$$)			
F.3	20	21	1.29	28.0	5.2	0.59	0.32	0.97	0.18
W.10	20	25	1.91	12.7	7.6	1.91	1.58	2.60	0.66
V.18	20	25	2.80	12.7	11.2	2.82	2.40	3.70	0.98

^a^$\hbox {PV}_{tot,est}$: estimated total pore volume: estimated from pedotransfer functions according to^50^ and corrected by stone content (see Eq. 1).

^b^TPS: total percolated biocide/tracer solution (L) after the percolation of four pore volumes.

^c^$\hbox {q}_{av}$ is the average water flux through the soil column and is the slope of the linear regression of the outflow (L) over time (h).

^d^$\hbox {q}_{min}$ and $\hbox {q}_{max}$ are the lowest and the highest calculated water flux during the column experiment.

^e^Calculated according to Eq. (2) with $\hbox {q}_{av}$ and a hydraulic head gradient of $3.2\,\hbox {cm}\,\hbox {cm}^{-1}$ (F.3) and $2.9\,\hbox {cm}\,\hbox {cm}^{-1}$ (W.10 and V.18).

The water flux decreased continuously with time in all soil columns: in W.10 and V.18 approximately 1.5 times and in F.3 approximately 3 times (Table 2, Figure S7 and Fig. S8). This could be due to clogging of pores in the $0.45\,\upmu \hbox {m}$ nylon membrane by colloids^51^ or dissolved organic matter^52^. In our experiment, the existence of colloids was likely as they could be mobilized by a decreased ionic strength of the infiltrating deionized water^53^.

#### Tracer breakthrough

**Figure 2:**
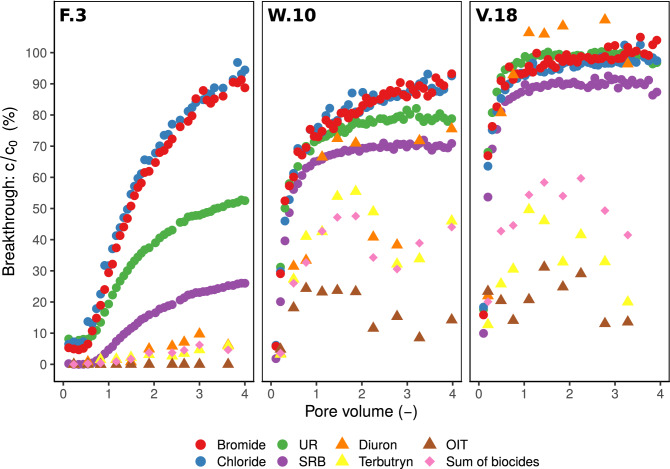
Breakthrough curves (BTCs) of the tracers bromide, chloride, UR, SRB and the biocides diuron, terbutryn, OIT and the sum of biocides (diuron+terbutryn+OIT; represented by diamonds). The normalized concentrations c/$\hbox {c}_0$ (%) in the outflow from soil columns was plotted against the pore volume (–).

Breakthrough curves (BTCs) of $\hbox {Br}^{-}$ and $\hbox {Cl}^{-}$ reached 90–100% at the outlet of all soil columns after percolation of four pore volumes (PV) (Fig. 2). Both ions showed the fastest breakthrough of all tracers suggesting only a weak interaction with the soil matrix; as in other studies, they can be considered as conservative tracers^54, 55^. Nevertheless, in our experiment, this assumption was challenged by the observation in the youngest SIS (F.3): after the percolation of one PV, only 30% $\hbox {Br}^{-}$ and $\hbox {Cl}^{-}$ broke through. Under the assumption that the transport of $\hbox {Br}^{-}$ and $\hbox {Cl}^{-}$ is only determined by convection, diffusion and dispersion, 50% of the solutes should have reached the outlet after one PV was exchanged^56^. The 20% deficit indicated that weak adsorption of $\hbox {Br}^{-}$ and $\hbox {Cl}^{-}$ occurred in F.3 as was also observed by other researchers, e.g.^57^. In contrast, at W.10 and V.18 substantially more than 50% $\hbox {Br}^{-}$ reached the outlet after one PV, indicating fast transport of $\hbox {Br}^{-}$ in macropores. However, since the PV of the individual soil columns are only rough estimates, they are subject to uncertainty. Therefore, the deviation from 50% solute breakthrough at a PV equals one could also be partly explained by the uncertainties in the calculation of the PV.

With the exception of UR at V.18, BTCs of UR and SRB did not reach 100% after four PV. Instead, their breakthrough maxima were $51\,\pm \,2\%$ (F.3) and $79\,\pm \,1\%$ (W.10) for UR; and $25\,\pm \,1\%$ (F.3), $70\,\pm \,1\%$ (W.10), and $90\,\pm \,2\%$ (V.18) for SRB. Maxima of BTCs at F.3 and W.10 increased in the following order: $\hbox {SRB}< \hbox {UR} <\hbox {Br}^{-} \approx \hbox {Cl}^{-}$. At V.18, this order slightly differed ($\hbox {SRB} < \hbox {UR} \approx \hbox {Br}^{-} \approx \hbox {Cl}^{-}$). These results indicated a stronger retention of the fluorescent tracers compared to $\hbox {Br}^{-}$ and $\hbox {Cl}^{-}$ most likely due to their higher adsorption affinity^58^. The higher retention of SRB compared to UR was in accordance with literature^59, 60^ and linear sorption coeffients ($\hbox {K}_d$-values) for SRB that were about twice as high compared to UR (Table S1 and Figure S11) for all SIS. However, the high discrepancy between the retention of conservative tracers ($\hbox {Br}^{-}$, $\hbox {Cl}^{-}$) and UR at F.3 revealed that UR ought to be considered as a non-conservative tracer with respect to its sorption properties. This was described previously for UR application in soils with comparable OC content, texture and pH^44, 61, 62^.

We observed a faster breakthrough of all substances in W.10 and V.18 than in F.3 as indicated by higher slopes and higher maxima of BTCs (Fig. 2). After the percolation of one PV, 29% (F.3), 73% (W.10), and 93% (V.18) of the $\hbox {Br}^{-}$ inflow concentrations were reached. Due to the small differences between the chemical soil properties of the SIS and the Kd values of UR and SRB (Table S1), we would have expected similar tracer BTCs. Yet, this was not the case; tracer retention decreased with age from the youngest to the oldest SIS (F.3 $<<$ W.10 < V.18). These differences could not be explained by chemical soil properties alone. When they would dominate solute transport, sorption, and thus solute retention should be highest at V.18 where OC content (1.9%) and clay content (8.8%) were highest and pH was lowest (7.1) (Table 1). From this we deduce that solute retention in W.10 and V.18 was controlled less by sorption than by preferential flow in macropores. Existing studies support this finding as they related fast transport of SRB^42, 43^ and UR^44^ in undisturbed soil cores to the existence of preferential flow, too. Furthermore, our results imply the following: when preferential flow in macropores dominates water and solute transport, the differences in the BTCs of substances with different adsorption affinities decrease. This effect led to the largest differences between tracer BTCs in F.3, where matrix flow was dominant, and to the smallest differences between tracer BTCs in V.18, where preferential flow was dominant. In F.3, the matrix flow was high and solutes had a longer contact to a larger number of sorption sites thus intensifying sorption losses. In V.18, the opposite was true: the contact of the solutes to sorption sites was brief as they bypassed the soil matrix with the preferential flow. Additionally, the number of sorption sites may be lower in the preferential flow regions^42^. Furthermore, similar BTCs of conservative and non-conservative tracers with different adsorption affinities indicate strong transport in macropores by preferential flow^63–65^.

#### Brilliant blue staining

**Figure 3:**
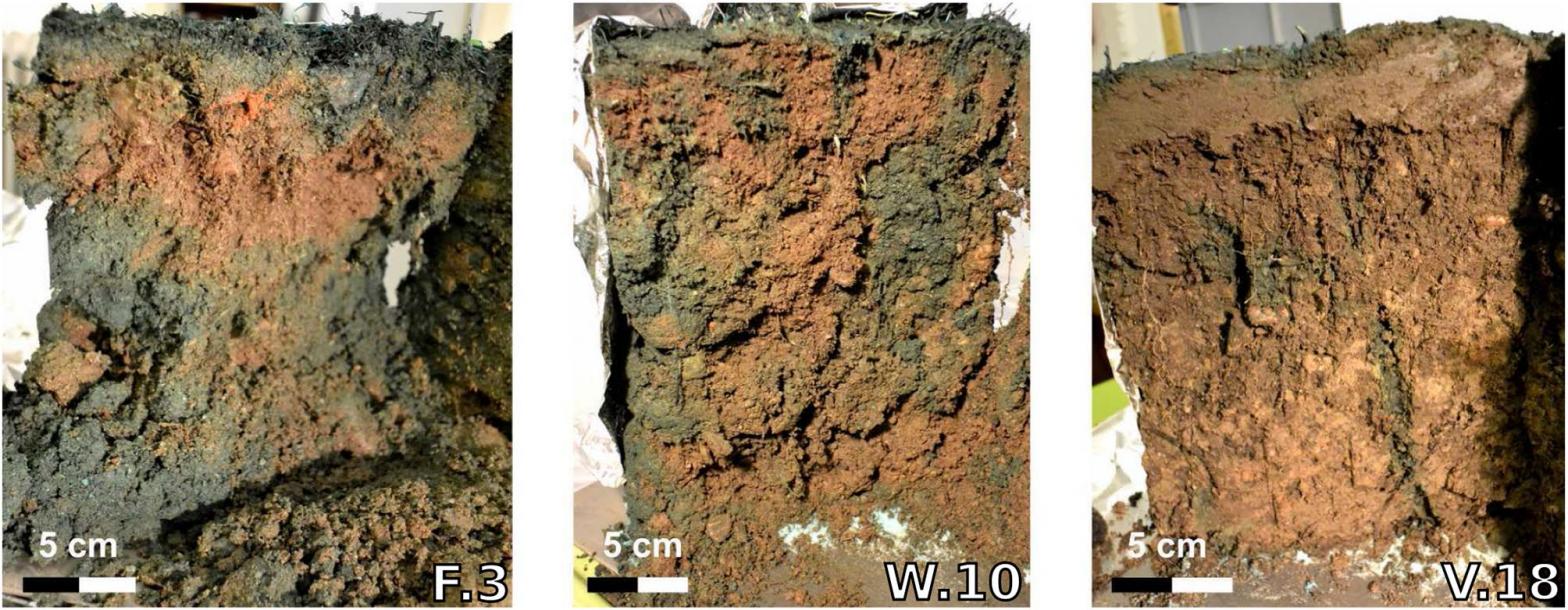
Brilliant blue stained soil columns (F.3, W.10 and V.18).

Roughly 65% of F.3, 38% of W.10 and 8% of V.18 (Figure S10) were stained. Along the cut profile large blue patches were visible for F.3 while only single blue fingers were observable for V.18 (Fig. 3). These macropores, especially in V.18, were most probably caused by earthworms^45, 66^. In fact, approximately ten individuals were observed on the laboratory columns after saturation from bottom to top. Earthworm holes were visible on the soil surface as well as on horizontal and vertical cuts through the soil column (approximately 2–3 mm, Figure S9). At W.10 several ants were observed that also could cause macropores^67, 68^. In contrast, no macrofauna was visible at F.3. Overall, brilliant blue patterns supported the assumption of an increasing fraction of preferential flow in the older SIS W.10 and V.18.

Brilliant blue staining also revealed partial water flow on the soil column sidewall^69^ that bypassed a part of the soil column F.3 and entered the soil again further down (Fig. 3). This partial sidewall flow could potentially explain the fast initial $\hbox {Br}^{-}$, $\hbox {Cl}^{-}$ and UR breakthrough in F.3 where 5–10% of the tracers was measured in the first sample taken after 30 min (Fig. 2). However, this effect was limited since concentrations remained only initially constant and increased again after approximately 0.5 PV. Interestingly, this effect could not be observed for the stronger adsorbing SRB. Possibly, SRB was initially retained and therefore could not be measured in the first percolates. Similar transport characteristics of UR and SRB in the beginning of the experiment were observed e.g. in the study conducted by^70^.

#### Biocide breakthrough

At all sites, the biocide breakthrough increased in the following order: OIT < terbutryn < diuron. This order followed the polarity of the substances as indicated by the $\hbox {K}_{OW}$-values (Table 3) which can be interpreted as a rough estimate for the sorption affinity of non-polar substances to organic matter^71, 72^. Furthermore, except for diuron at W.10 and V.18, biocides were more strongly retained than the tracers in all SIS. This observation may be explained as follows: the walls of macropores caused by earthworms may be enriched with soil organic matter^73^. These macropores may provide hydrophobic sorption sites (organic matter) for non-polar substances such as biocides and less for the polar tracers.

Analogous to UR and SRB, the retention of biocides was highest in F.3 (Fig. 2), followed by W.10 and V.18 and was negatively related to the OC content of all SIS (Table 1). Similar to the tracers, the large differences in BTCs of biocides between the SIS cannot be explained by chemical soil properties but rather by soil structure. In F.3, biocides had more intense contact with the soil matrix than in W.10 and V.18 due to a higher fraction of matrix flow. As a result, biocide retention was highest in F.3 despite the lowest OC content. Thus, the influence of biocide properties on their retention was strongly reduced by preferential flow in macropores^74, 75^.

Although biocide degradation is unlikely due to the short duration of the experiment (approximately 13–28 h) and due to immediate cooling and freezing of the samples after sampling, these processes could not be completely ruled out. However, if a small amount of biocide degradation occurred, then concentrations would have been reduced by the same amount in all samples. The differences in biocide concentrations between the individual soil columns should not have been affected.

The rapid leaching of organic pollutants through macropores, has also been observed in several studies for pesticides in agricultural soils^38, 39^. More biocide breakthrough in W.10 and V.18, especially that of diuron in V.18 is in line with findings of^76^, who reported leaching of diuron due to complexation of diuron with dissolved organic matter (DOM). This indicates that DOM molecules have a dual role on pollutant transport in soils: they compete for adsorption of the organic pollutants and enhance their mobility by complexation. But their leaching is enhanced by preferential transport in macropores following intense rainfall^77^ or in strongly structured soils with connection to shallow groundwater^78^.

### Important factors of fast solute breakthrough in urban SIS and implications for storm water treatment

Despite similar chemical soil properties between the SIS, W.10 and V.18 showed a much faster tracer and biocide breakthrough compared to F.3. Brilliant blue patterns and saturated hydraulic conductivities indicated prevailing preferential flow conditions in macropores in SIS W.10 and V.18 that lead to fast solute breakthrough. Macropores can be caused by biological activity^41, 79^, which is supported by the observation of the highest activity of macrofauna (earthworms and ants) in the older SIS W.10 and V.18. Therefore, in our experiment the age of SIS is closely related to macrofauna activity. This observation is in agreement with results from^80^ who found an increasing earthworm population density with increasing age of different urban landscapes. Similar observations were made by^81^ in technosols but they concluded that the existence of an initial addition of topsoil is required, which was the case in all SIS of our study.

Beside the age of a soil, the abundance of earthworms is also controlled by bulk density and soil depth^82^. In SIS V.18 bulk density was lowest and soil depth highest, both factors favoring high earthworm abundance. Thus, different relevance of preferential flow in the different SIS may also be due to differences in the construction of the SIS. If a SIS is built with a less thick topsoil layer or a high bulk density, or if other macrofauna-inhibiting factors play a role, the development of the macrofauna may be restricted, making the development of preferential flow paths in aging SIS less likely. Furthermore, climate can influence the population dynamics of earthworms^83, 84^. Therefore, we conclude that factors supporting higher activity of macrofauna (e.g. earthworms and ants) in SIS may also lead to faster solute breakthrough.

Additional influence on macropore formation can be exerted by the vegetation of the swales, directly by plants with thicker or deeper roots colonizing as development of the swales progresses, or indirectly by influencing the diversity of invertebrates^85^. Due to necessary time for colonization and establishment of species also the age of swales may be linked to the biodiversity. In addition to vegetation and soil structure, the shape of the swales could also have an influence on diversity of invertebrate and thus on macropore formation. Reference^85^ supposed that biodiversity may be higher in round systems than in linear ones. However, we observed rather the opposite, suggesting that age played a greater role for macrofauna diversity and macropore formation than swale shape in our experiments.

Our experiment has shown that SIS can retain biocides by adsorption when the substances have sufficient contact with the soil matrix. Inversely, our study also suggests a decrease in biocide retention capacity in urban SIS due to preferential flow pathways caused by an increasing biological activity and changing soil properties already after 10–18 years of operation. However, biological activity in SIS is also desirable due to several benefits. Higher biodiversity of vegetation and soil fauna may enhance degradation of several organic pollutants and higher infiltration rates, e.g. due to a higher number of macropores, may guaranty fast water infiltration even after long-term operation. Overall, we recommend regular monitoring of the pollutant retention capacity of SIS to detect its reduction in time, which could be done, for example, by tracer experiments.

One approach to address this problem is to treat storm water with special adsorbent materials^86, 87^ before it enters the swales, or integrate additional adsorbent layers in the swales. However, these options are complex and expensive and, furthermore, a large portion of urban runoff often infiltrates diffusely and does not reach the swales at all. Therefore, a more sustainable approach, is to avoid biocide pollution at the source^88, 89^, which would allow a targeted urban water management by SIS that preserves urban groundwater quality.

## Methods

### Study sites and soil characterization

We selected three SIS of different age (F.3, W.10 and V.18) in the city of Freiburg, south-west Germany (Figure S1). F.3 (3 years old) was a nearly rectangular shaped swale (approx. $600\,\hbox {m}^{2}$) that drained a commercial area, W.10 (10 years old) a rectangular multilevel swale system (approx. $700\,\hbox {m}^{2}$), and V.18 (18 years old) an elongated swale-trench system (approx. $3000\,\hbox {m}^{2}$), both drained a residential area. F.3 consisted of a 25–30 cm topsoil layer over the natural soil layer. Beneath a 30–50 cm topsoil layer of W.10 and V.18 there was a 20 cm sand layer followed by a gravel-filled drain trench to collect and drain the seepage water. To ensure comparability despite different SIS geometry, all samples were taken in the intermediate vicinity of the inflow and from similar depths (20–25 cm). Moreover, the inflow area is particularly interesting because it represents the entry point of pollutants in the SIS. The vegetation of the swales consisted mainly of grass as well as clover (*Trifolium*), dandelion (*Taraxacum*) and ribwort (*Plantago*).

From each SIS, four fixed volume soil cores (diameter: 8 cm, length: 15 cm) were taken with a root auger in a 2 $\times$ 2 m square at two depths (0–15 cm and 15–30 cm). This was performed in two steps (Figure S2): after taking the upper soil core (0–15 cm), the deeper one was taken from the same bore hole (15–30 cm).

Using a knife, each soil core was cut into 5 cm wide pieces resulting in five (F.3, W.10) and six (V.18) depth-related soil samples (0–5 cm, 5–10 cm, 10–15 cm, 15–20 cm, 20–25 cm, 25–30 cm; $\hbox {n} = 4$). Soil samples were air-dried and weighed to calculate bulk density. Subsequently, the soil samples passed through a 2 mm sieve and stones (inorganic particles $> 2\,\hbox {mm}$) and roots were weighed separately. We determined the residual gravimetric water content as the difference between the weight of an air-dried soil sample before and after 24 h of drying at $105\,^{\circ }\hbox {C}$. The pH was measured from over-night, 25 ml 0.01 M $\hbox {CaCl}_{2}$ supsensions of 10 g soil at room temperature ($23\,^{\circ }\hbox {C}\,\pm \,2\,^{\circ }\hbox {C}$) with a pH meter (Deutsche METROHM GmbH & Co KG, Filderstadt, Germany). The content of sand, silt and clay (inorganic particles $<2\,\hbox {mm}$; fine soil) were determined by sieving, sedimentation and the pipette method^90^. The OC content was determined by a CNS-analyzer (vario EL cube, Elementar Analysensysteme GmbH, Germany). Due to the possible carbonate content ($\hbox {pH} > 7.0$), the OC content of the soil samples was determined as the difference of total carbon before and after heating at $550\,^{\circ }\hbox {C}$. At this temperature OC had been transformed to $\hbox {CO}_{2}$^91^. Additionally, sorption isotherms for UR and SRB were produced according to OECD guideline 106^92^ described in^62^ and linear sorption coeffients ($\hbox {K}_{d}$-values) were calculated.

### Percolation experiment

In every SIS, one intact soil column was collected using a stainless steel cylinder (diameter: 20 cm, length: 30 cm) that was knocked into the soil. The cylinder was excavated and the embedded soil column was pushed into a second steel cylinder (Figure S3). The bottom of the soil column was straightened with a knife and approx. 1–2 cm sand was added to ensure connection to a $0.45\,\upmu \hbox {m}$ nylon membrane. The height of soil column F.3 was lower (21 cm) than of W.10 and V.18 (25 cm) because the soil layer of F.3 was shallower. During sampling, the vegetation was left on the soil column as far as possible. Where it was too long, it was superficially shortened. Plants were not removed so as not to disturb the soil structure.

The top of the soil column was connected to two storage vessels (Figures S5, S6). The first storage vessel was connected via a tube with a small liquid layer on the top of the soil (approximately 2 cm). The second storage vessel was connected to the first one with two tubes that kept the water level between the storage vessels and the soil column constant. At the bottom of the soil column, the solution was placed under tension with a 45 cm water head to quicken flow rates and thus reduce anaerobic conditions in the soil column.The bottom of the column was enclosed with a $0.45\,\upmu \hbox {m}$ nylon membrane. The percolate was collected in 1 L-glass bottles that were placed on weighing devices (Figure S5) to calculate the flow rate. To avoid photodegradation, all vessels, tubes and bottles were wrapped with aluminum foil. The soil columns were saturated with deionized water from bottom to top at $7\,\hbox {cm}\,\hbox {d}^{-1}$. Thereafter, deionized water was percolated to reach constant flow conditions and to decrease the DOC load of the percolate. Pre-tests with $\hbox {Br}^{-}$ (NaBr, Carl Roth GmbH & Co KG, Karlsruhe, Germany) were conducted to set up a sampling protocol for each soil column. Before starting the main experiment, $\hbox {Br}^{-}$ was washed out by deionized water.

The main percolation experiment started with a 1.5 h flushing of the soil columns with deionized water. Subsequently, all water in the storage vessels and on the soil surface was replaced by a tracer/biocide solution. For each soil column, 15 L of initial solution consisting of deionized water, tracers and biocides were prepared. Target tracer concentrations were $50\,\hbox {mg}\,\hbox {L}^{-1}\ \hbox {Br}^{-}$, $25\,\hbox {mg}\,\hbox {L}^{-1}\ \hbox {Cl}^{-}$ ($\hbox {CaCl}_{2}$, VWR International GmbH, Darmstadt, Germany), $10\,\hbox {mg}\,\hbox {L}^{-1}$ UR (Simon & Werner GmbH, Flörsheim, Germany), $400\,\hbox {mg}\,\hbox {L}^{-1}$ SRB (Chroma GmbH & Co KG, Münster, Germany). Target biocide concentrations are based on commonly measured concentrations in facade runoff^93^ and were $50\,\hbox {mg}\,\hbox {L}^{-1}$ each of diuron, terbutryn (NEOCHEMA GmbH, Bodenheim, Germany) and OIT (Sigma-Aldrich Chemie GmbH, Taufkirchen, Germany). The tracer stock solutions were prepared with deionized water and solid substances, while the biocides were already dissolved in acetonitrile by the manufacturer. UR and SRB solutions were stored in amber glass bottles and wrapped with aluminum foil to prevent photolytic decay.

The measured initial concentrations only slightly deviated from the intended concentrations (Table S5). The experiment lasted for 28 h (F.3) and 12.7 h (W.10, V.18) depending on the flow velocity. We excluded biodegradation of biocides since their half-times in soils are much higher than the duration of the experiment^29^. Furthermore, terbutryn, diuron and OIT are assumed to be stable to aqueous hydrolysis^28, 94^. In the beginning of the experiment, samples of the percolate were taken every 15 min, later every 20, 30, 45 or 60 min. An aliquot of the collected percolate was filled into 100 mL amber glass bottles for UR, SRB and biocide measurements, and 100 mL polyethylene bottles for $\hbox {Br}^{-}$, $\hbox {Cl}^{-}$ and pH measurements. Samples were stored at approximately $6^{\circ }\hbox {C}$ for measurement for a maximum of ten days and frozen for longer storage. No changes in concentrations were observed in preliminary laboratory tests measuring biocide concentrations before and after storage (freezing of samples for multiple weeks).

The estimated total pore volume $\hbox {PV}_{tot,est}$ (L) of each soil column (Table 2) was calculated by the following:

1 $$\begin{aligned} PV_{tot,est} (L) = \text {porosity} \, (-) \cdot \left( 1 - \left( \frac{\text {stone content}\ (\%)}{100\%}\right) \right) \cdot \text {total volumn of the soil column (L)}. \end{aligned}$$

The porosity (–) was estimated according to^50^, who provide average porosities for soils in dependence of their texture and OM content. The estimated water volume that flowed through the column at time t ($\hbox {PV}_{t}\ \hbox {L}\,\hbox {L}^{-1}$) was calculated by dividing the outflow (L) at a certain time step by $\hbox {PV}_{tot,est}$. $\hbox {PV}_{t}$ was used to normalize the percolated amount of water and make solute transport comparable. Maxima of BTCs of the solutes were estimated by calculating the mean breakthrough (%) between PVs of three and four. The saturated hydraulic conductivity $\hbox {K}_{s}$ ($cm\,\hbox {h}^{-1}$) was calculated according to Darcy’s law:

2 $$\begin{aligned} q = K_s \cdot \frac{\Delta H}{L}. \end{aligned}$$

where q ($cm\,\hbox {h}^{-1}$) is the water flow through the soil column, $\Delta H$ (cm) is the hydraulic head difference between upper and lower boundary of the soil column, and L (cm) is the length of the column. Data analysis was performed with R statistics (version 3.3.4)^95^.

### Brilliant blue staining

To identify preferential flow in soils of SIS we used brilliant blue staining as was done before e.g. by^42, 96^. In each soil column about 3.5 L (approximately one PV) brilliant blue FCF (Waldeck GmbH & Co KG, Germany) solution ($c=2\,\hbox {g}\,\hbox {L}^{-1}$) was applied. Due to different flow velocities, it took 23 h (F.3), 8.2 h (W.10) and 5.2 h (V.18) until the brilliant blue solution infiltrated. Subsequently, we removed the soil column from the steel cylinders, cut them in half using a knife and photographed them (Canon 450D) to make flow pathways visible. Pictures were processed by Gimp 2.10 (The GIMP team, www.gimp.org) and ImageJ-win 64 (Fiji Is Just ImageJ, fiji.sc)^97^ (see Note S1). The correlation between the brilliant blue stained area of the soil columns and the breakthrough maxima was tested with Pearson correlation coefficients.

### Measurement of salt and fluorescent tracers

UR and SRB (Table 3) fluorescence was measured at 488 nm (UR) and 560 nm (SRB) in a synchronous scan method (wavelength range: $\lambda = 250$–650 nm, $\Delta \lambda = 25\,\hbox {nm}$) using the luminescence spectrometer LS-50B (Perkin Elmer, MA, USA). Due to sensitivity of tracer fluorescence to pH^98^, it was buffered before measurement at 9–10 using one drop of 1.5 M EDTA to ensure 100% fluorescence intensity for both tracers. To ensure a linear calibration range, the calibration solutions were prepared in the ranges 0.25–$5\ \upmu \hbox {g}\,\hbox {L}^{-1}$ (UR) and 5–$90\ \upmu \hbox {g}\,\hbox {L}^{-1}$ (SRB). The calibration of UR and SRB was performed separately for each SIS (extracting agent: 0.01 M $\hbox {CaCl}_{2}$ solution, soil:solution-ratio: 1:5). Samples for the measurement of UR and SRB were diluted 1:10 to reduce DOC background fluorescence and to maintain the calibration range. $\hbox {Br}^{-}$ and $\hbox {Cl}^{-}$ were measured by ion chromatography (790 Personal IC, Deutsche METROHM GmbH & Co KG, Filderstadt, Germany).

**Table 3:**
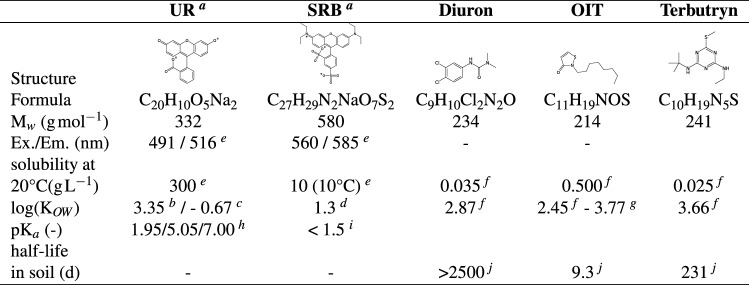
Characterisation of fluorescent tracers and biocides.

$^{a}$Color Index (Name:Number): UR (Acid Yellow 73 : 45350)^58^ ; SRB (Acid Rd 52 : 45100)^58^;

$^{b}$log($\hbox {K}_{OW}$) for the twofold protonated (neutral) species (dominant species between pH 1.95 and 5.05), Calculated with Estimation Programs Interface Suite for Microsoft Windows, v 4.11^99^;

$^{c}$log($\hbox {K}_{OW}$) for the disodium salt (twofold negative charged) species (dominant species above pH 7.00), Calculated with Estimation Programs Interface Suite for Microsoft Windows, v 4.11^99^;

$^{d}$log($\hbox {K}_{OW}$) at pH 7.15^100^;

$^{e}$Source:^58^; $^{f}$Source:^94^; $^{g}$Source:^101^; $^{h}$Source:^102^; $^{i}$Source:^103^; $^{j}$Source:^29^.

### Measurement of biocides

Analysis of terbutryn, diuron and OIT was already described in detail in^28, 104^. For measurement of biocides, 3 mL of the percolates were evaporated to dryness with a Büchi Syncore Polyvap (BÜCHI Labortechnik GmbH, Essen, Germany) and taken up in 0.3 mL acetonitrile (enrichment factor of 10). $90\,\upmu \hbox {L}$ of the percolate sample containing biocides were spiked with $10\,\upmu \hbox {L}$ of terbutryn-D5 as an internal standard. Measurements of percolate samples were conducted with a Triple Quadrupole mass spectrometer (Agilent Technologies, 1200 Infinity LC-System and 6430 Triple Quad, Waldbronn, Germany). Therefore, a NUCLEODUR RP-C18 column (125/2 100–$3\,\upmu \hbox {L}$ C18 ec; MACHEREY-NAGEL GmbH & Co KG, Düren, Germany) was used as stationary phase, whereas 0.01% formic acid (A) and acetonitrile (B) were used as mobile phases with a flow of $0.4\,\hbox {mL}\,\hbox {min}^{-1}$ and the following concentration gradient: 10% B (0–1 min), 10–50% B (1-11 min), 50–85% B (11–18 min), 85–90% B (18–21 min), 90% B (21–24 min), 90–10% B (24–26 min), 10% B (26–30 min). Oven temperature was $\hbox {T} = 30\,^{\circ }\hbox {C}$ and injection volume $5\,\upmu \hbox {L}$. Mass spectrometric settings are listed in Table S2. The linearity between peak area and concentration of substances were obtained in a range of 0–$5\,\upmu \hbox {g}\,\hbox {L}^{-1}$. Hence, limits of detection (LOD) and quantitation (LOQ) were calculated with DINTEST (2003) according to DIN 32645 and amounted to 1 and $2.65\,\upmu \hbox {g}\,\hbox {L}^{-1}$ (diuron), 0.76 and $2.94\,\upmu \hbox {g}\,\hbox {L}^{-1}$ (terbutryn), 0.86 and $2.99\,\upmu \hbox {g}\,\hbox {L}^{-1}$ (OIT), respectively.

## Supplementary Information

Supplementary Information 1

## Data Availability

All data generated or analyzed during this study are included in this published article (and its Supplementary Information files).
