# Supplementary material for: Urban storm water infiltration systems are not reliable sinks for biocides: evidence from column experiments
Source: Sci Rep. 2021 Mar 31;11:7242. doi: 10.1038/s41598-021-86387-9 (PMC8012575; doi:10.1038/s41598-021-86387-9)
Supplement: Supplementary file 1 — Supplementary Information 1 [file 41598_2021_86387_MOESM1_ESM.pdf]

Supplement to:
Urban storm water infiltration systems are not reliable sinks for biocides: Evidence from column experiments

Marcus Bork[1,2,*], Jens Lange[1], Markus Graf-Rosenfellner[2], Birte Hensen[3], Oliver Olsson[3], Thomas Hartung[2], Elena Fernández-Pascual[3,4], and Friederike Lang[2]

[1]Hydrology, Faculty of Environment and Natural Resources, University of Freiburg, Freiburg, 79098, Germany
[2]Soil Ecology, Faculty of Environment and Natural Resources, University of Freiburg, Freiburg, 79098, Germany
[3]Institute of Sustainable and Environmental Chemistry, Leuphana University Lüneburg, Lünbeburg, 21335, Germany
[4]Environmental Research Institute, University College Cork, Cork, T23 XE10, Ireland
[*]marcus.bork@hydrology.uni-freiburg.de

List of notes

1 Note S1: Picture editing . . . 3
2 Data . . . 14

List of Figures

Figure S1 Investigated storm water infiltration systems. . . . 4
Figure S2 Sampling design. . . . 5
Figure S3 Sampling of soil columns. . . . 6
Figure S4 Particle size distribution of SIS F.3, W.10 and V.18. . . . 7
Figure S5 Setting of the percolation experiment. . . . 8
Figure S6 Scheme of experimental setting of percolation experiment. . . . 9
Figure S7 Outflow (L) over time (h) of percolation experiment within the percolation of four pore volumes. . . . 10
Figure S8 Flux q over time (h) of percolation experiment. . . . 11
Figure S9 Earthworm-holes and preferential flow paths on the oldest soil column (V.18). . . . 11
Figure S10 Brilliant blue stained soil columns and imageJ pictures. . . . 12
Figure S11 Adsorption isotherms for UR and SRB. . . . 13

List of Tables

Table S1 K_d-values for UR and SRB determined in batch experiments. . . . 14
Table S2 Analytical parameter of the analysis of substances in LC-MS/MS. Fragmentation voltage (FV) and collision energy (CE) of the first and second transition. . . . 14
Table S3 Total mean depths of soil cores. . . . 14
Table S4 Repetitions of soil property analyses. . . . 15
Table S5 Target and actual initial concentrations for tracers and biocides. . . . 16
Table S6 Depth-dependent soil properties (data to Fig. 1; means and standard diviations; repetitions are shown in Table S4). . . . 16
Table S7 Data of the soil column experiment (data to Fig. 2). Outflow: Amount of water at the bottom of the soil column at each time step; $c_{Outflow}$: concentration in the outflow at each time step; PV: pore volume. . . . 17
Table S8 Mean concentrations and standard deviations (n = 3) of adsorption isotherms (data to Fig. 3). . . . 29

Note S1: Picture editing

The photos of cut surfaces were processed using the image processing program Gimp 2.10 (The GIMP team, www.gimp.org). First, the soil column was cut out using the magnetic scissor tool. Following, the clearly blue coloured surfaces were extracted using the pipette function and were replaced by black colour. Then, the processed picture was loaded into the image analysis program ImageJ-win 64 (Fiji Is Just ImageJ, fiji.sc)[1]. In this program, the fraction of black area on the whole area of soil monolith was determined. The image manipulation with Gimp (change brilliant blue colour to black colour) was necessary to increase the selectivity of the analysis program, which works with 256 greyscales.

Literature:

[1] Schindelin, J. et al. Fiji: An open-source platform for biological-image analysis. Nature methods 2012, 9, 676-682.

Figures

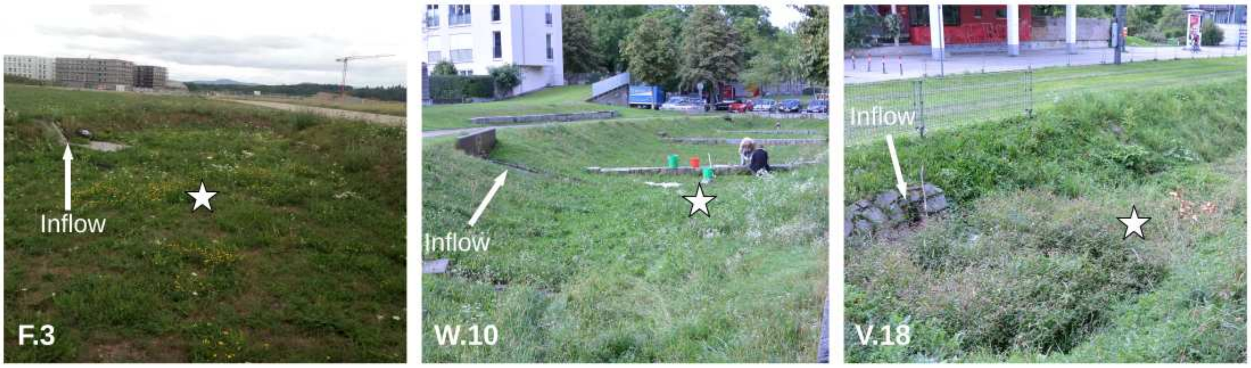

Figure S1. Investigated storm water infiltration systems (F.3, W.10 and V.18). The sampling points (white stars) were placed near the SIS inflows.

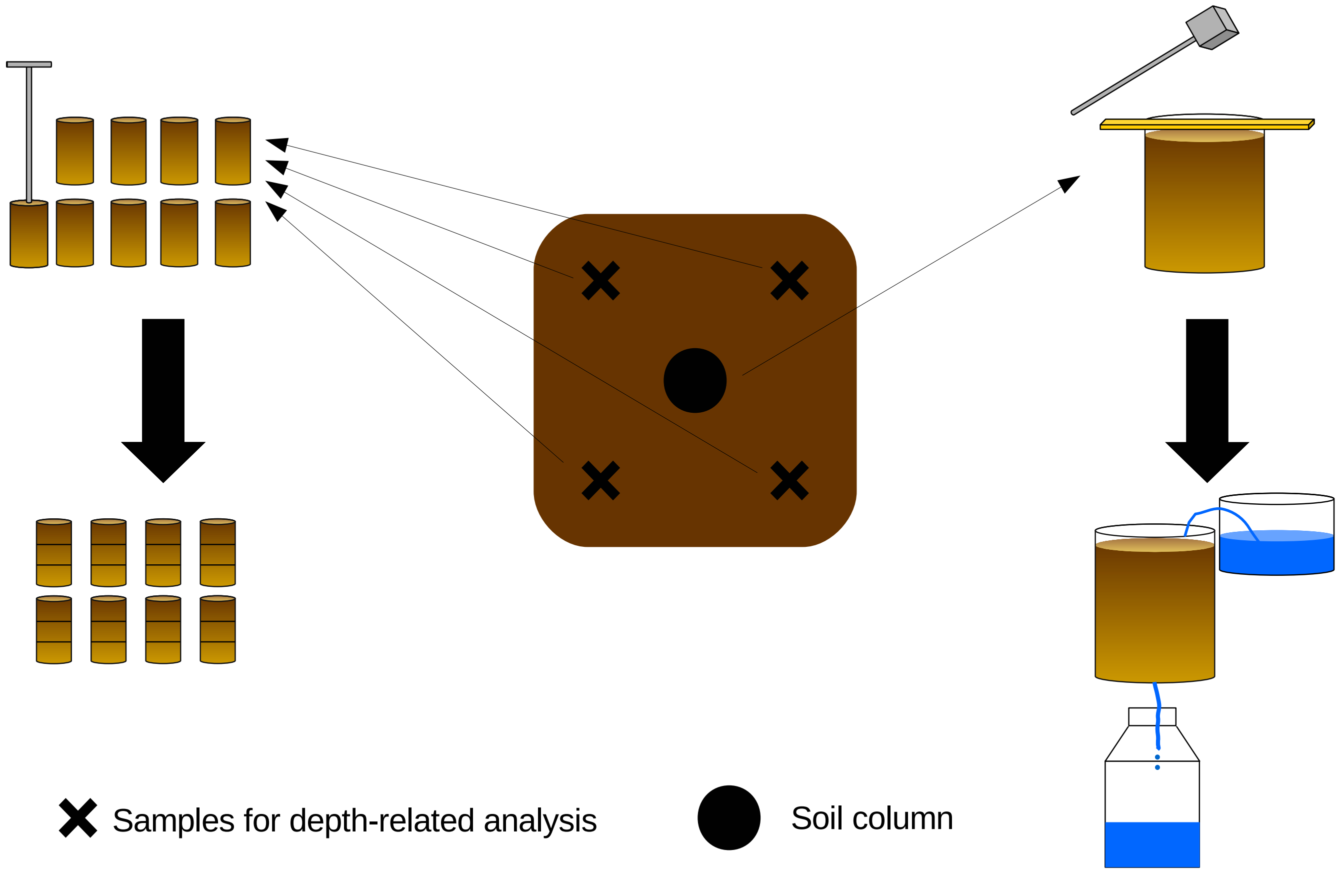

Figure S2. Sampling design. At each site (F.3, W.10, V.18) four soil cores (diameter: 8 cm, length: 15 cm) were taken in two depths (0-15 cm and 15-30 cm) and were cut in 5 cm wide slices for depth-related analysis. Additionally, at each site one soil column (diameter: 20 cm, length 25-30 cm) was taken.

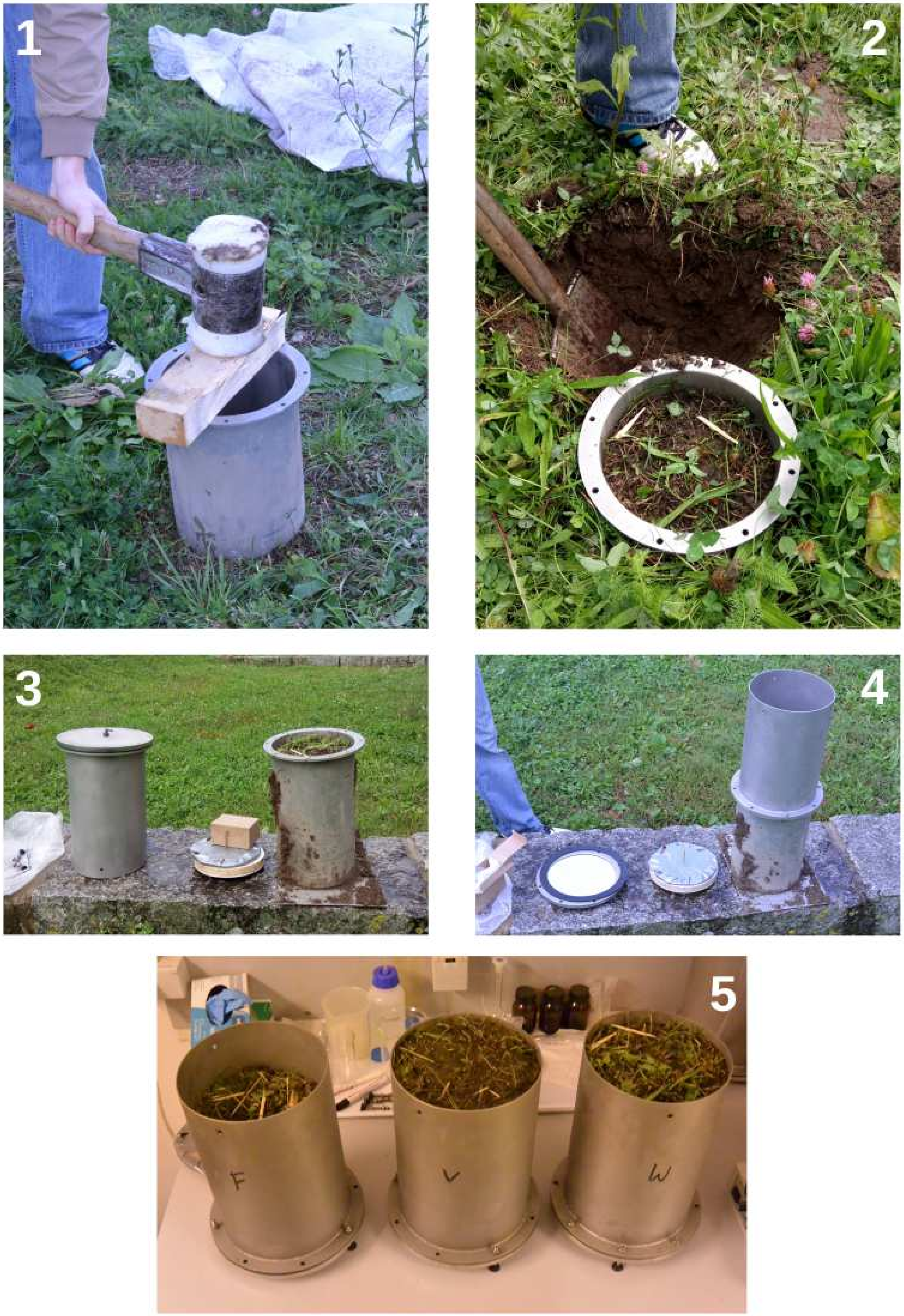

Figure S3. Sampling of soil columns.

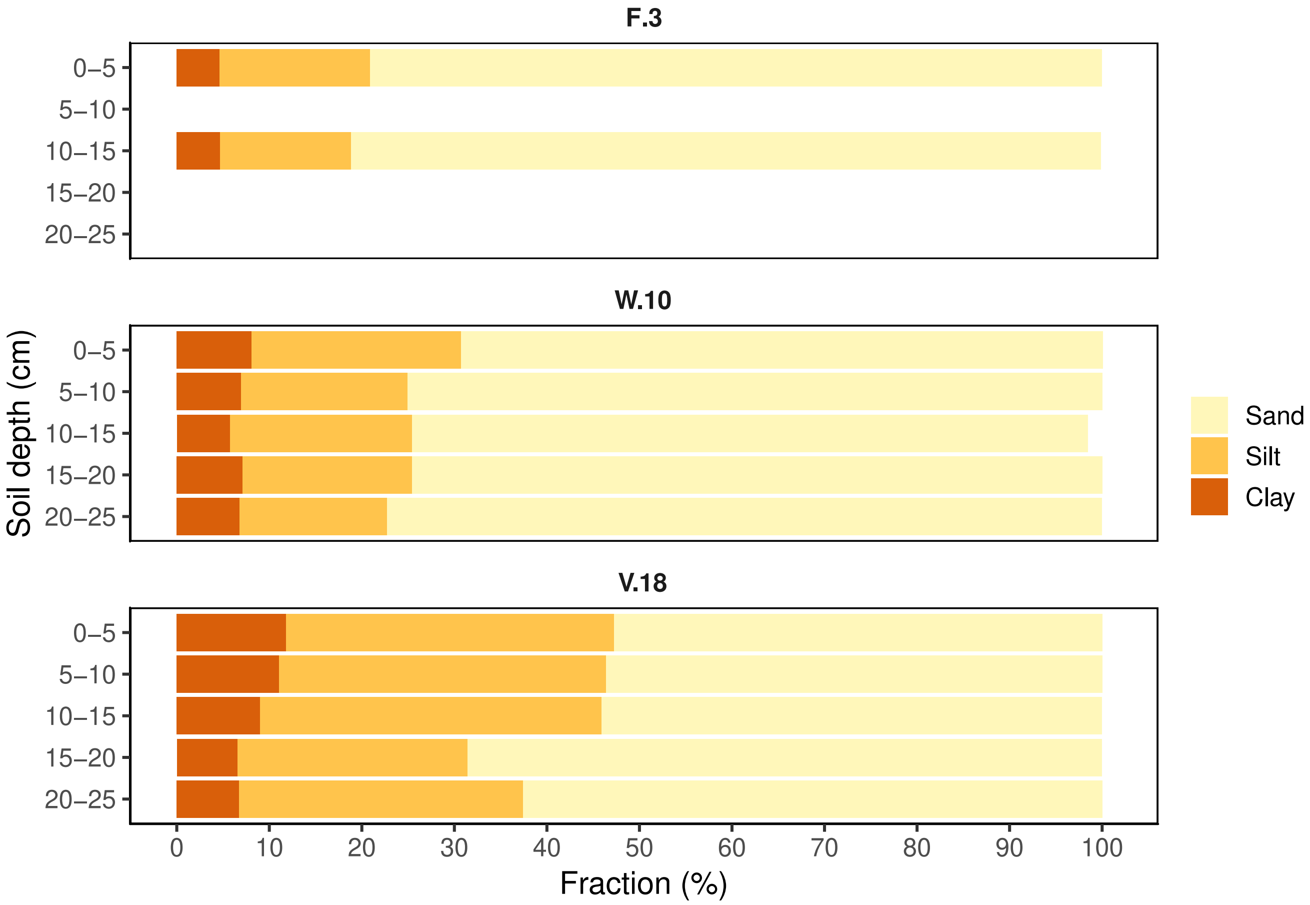

Figure S4. Particle size distribution of SIS F.3, W.10 and V.18.

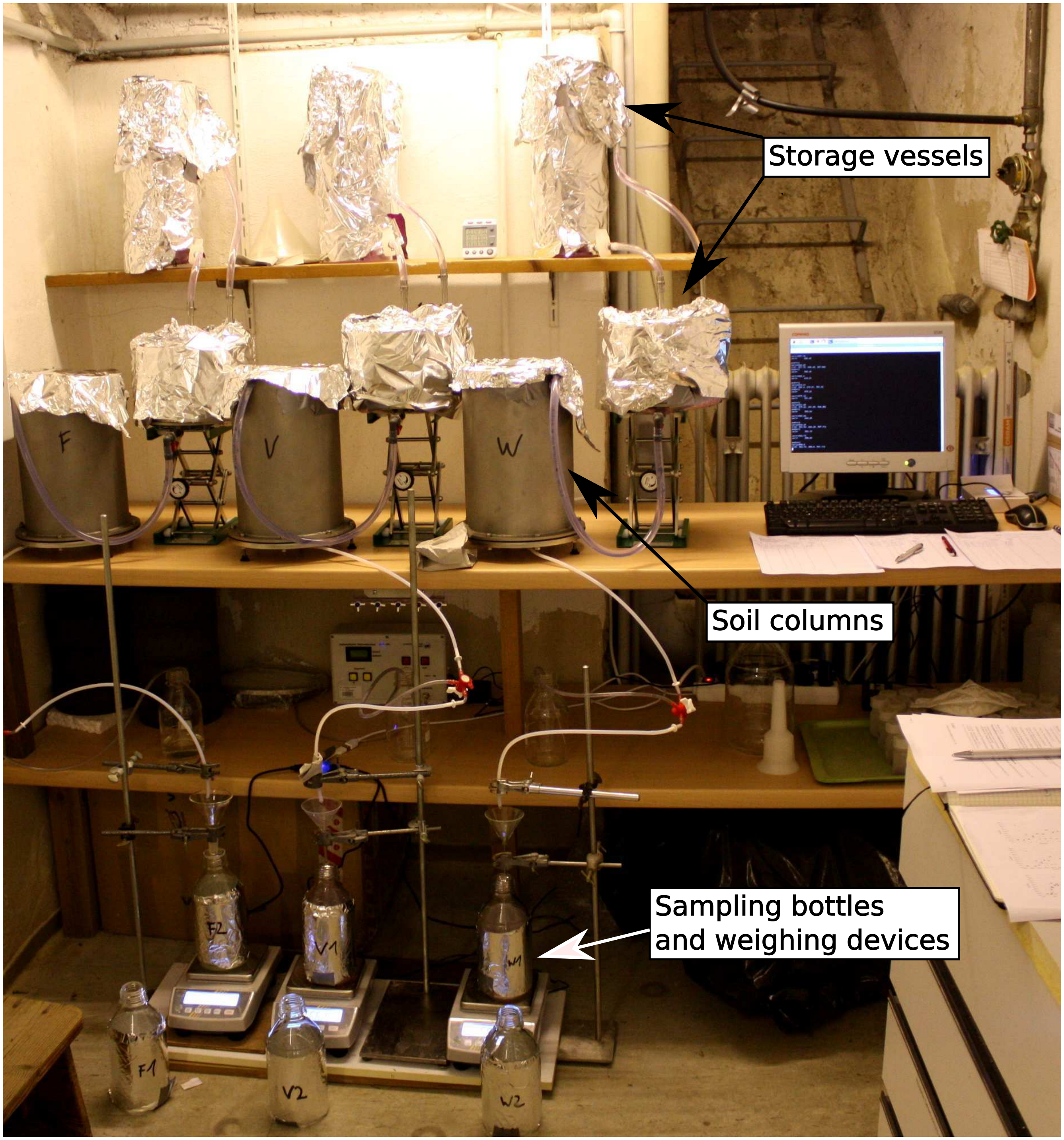

Figure S5. Setting of the percolation experiment.

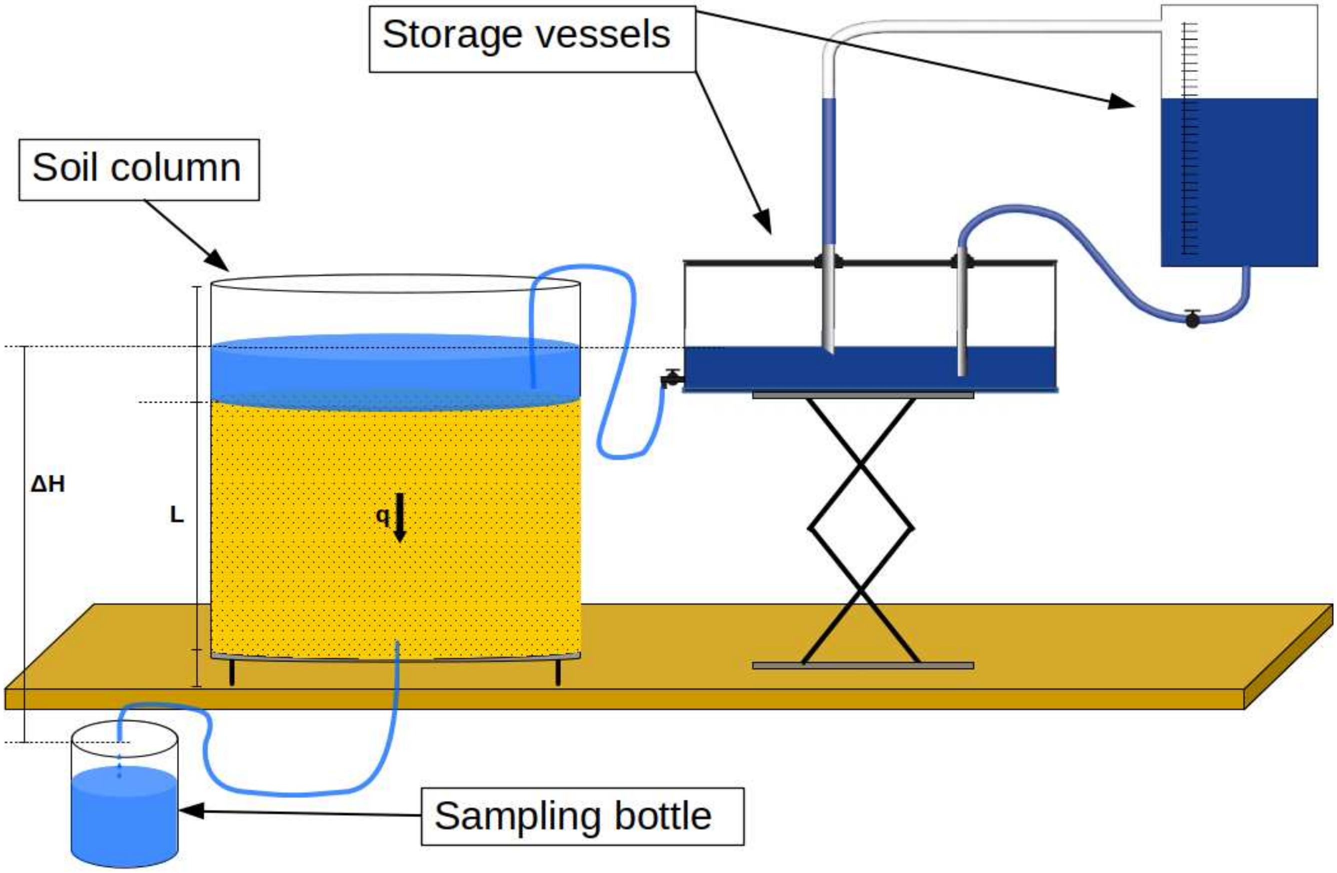

Figure S6. Scheme of experimental setting of percolation experiment. q: flux density; L: length of soil monolith; ΔH: hydraulic head difference.

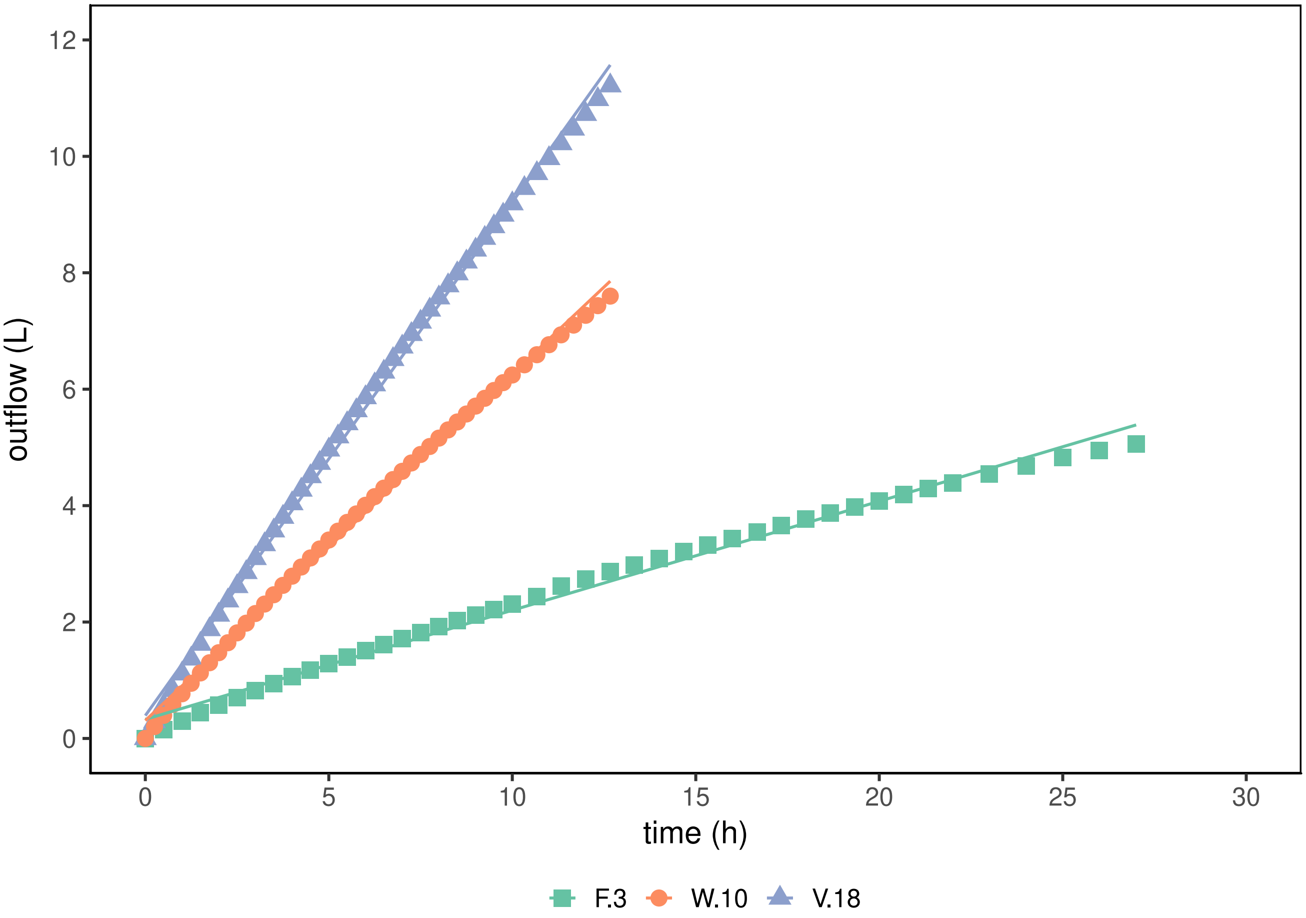

Figure S7. Outflow (L) over time (h) of percolation experiment within the percolation of four pore volumes.

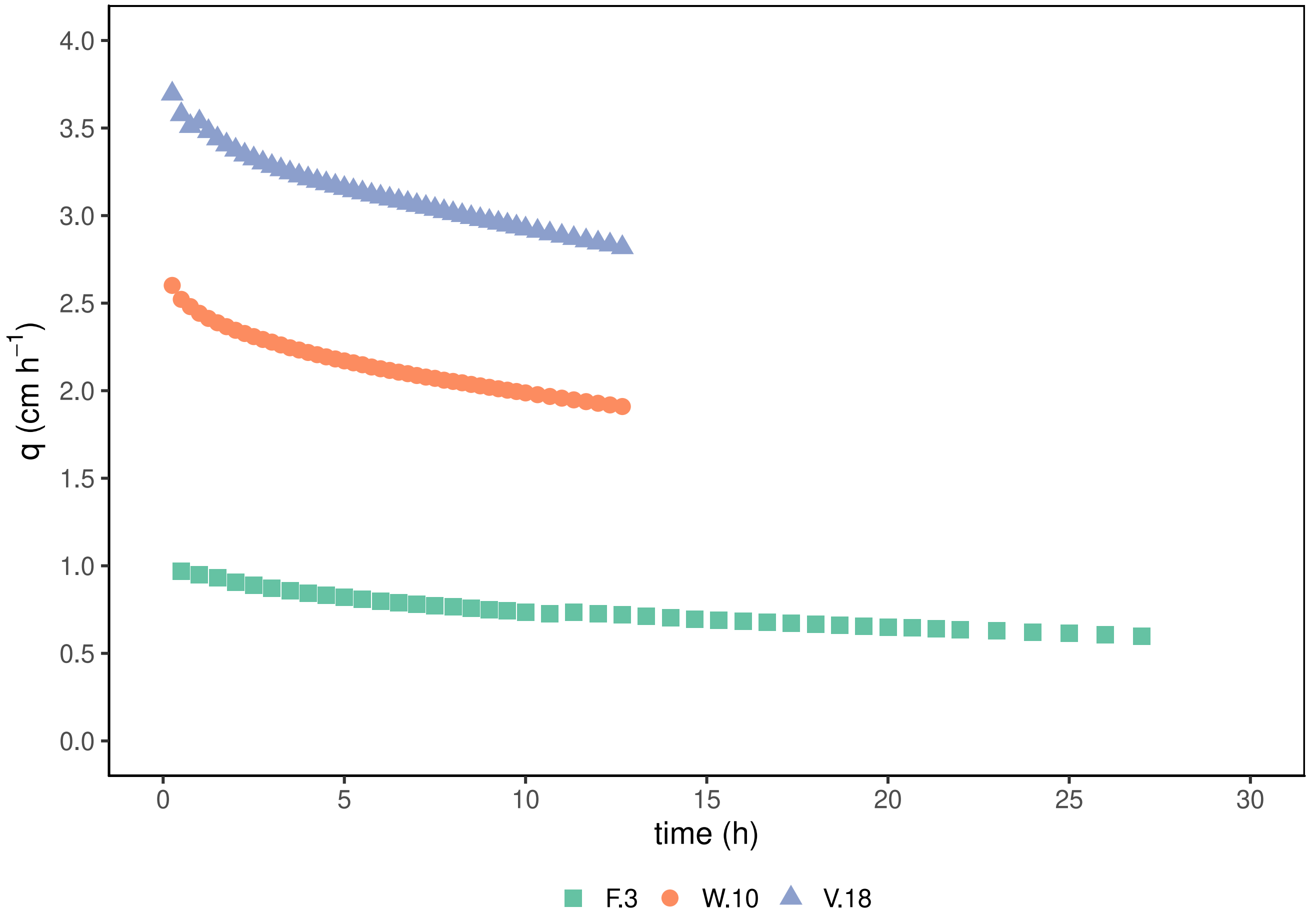

Figure S8. Flux q over time (h) of percolation experiment.

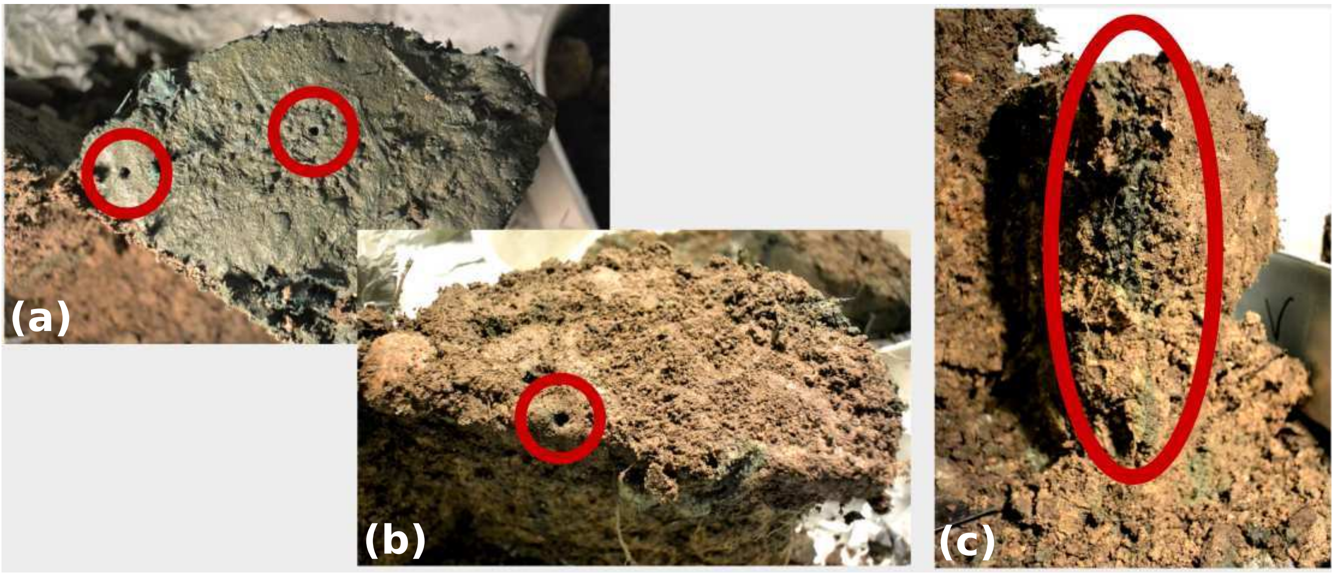

Figure S9. Earthworm-holes and preferential flow paths on the oldest soil column (V.18). (a) surface of soil column; (b) horizontal cut through soil column; (c) vertical cut through an earthworm hole.

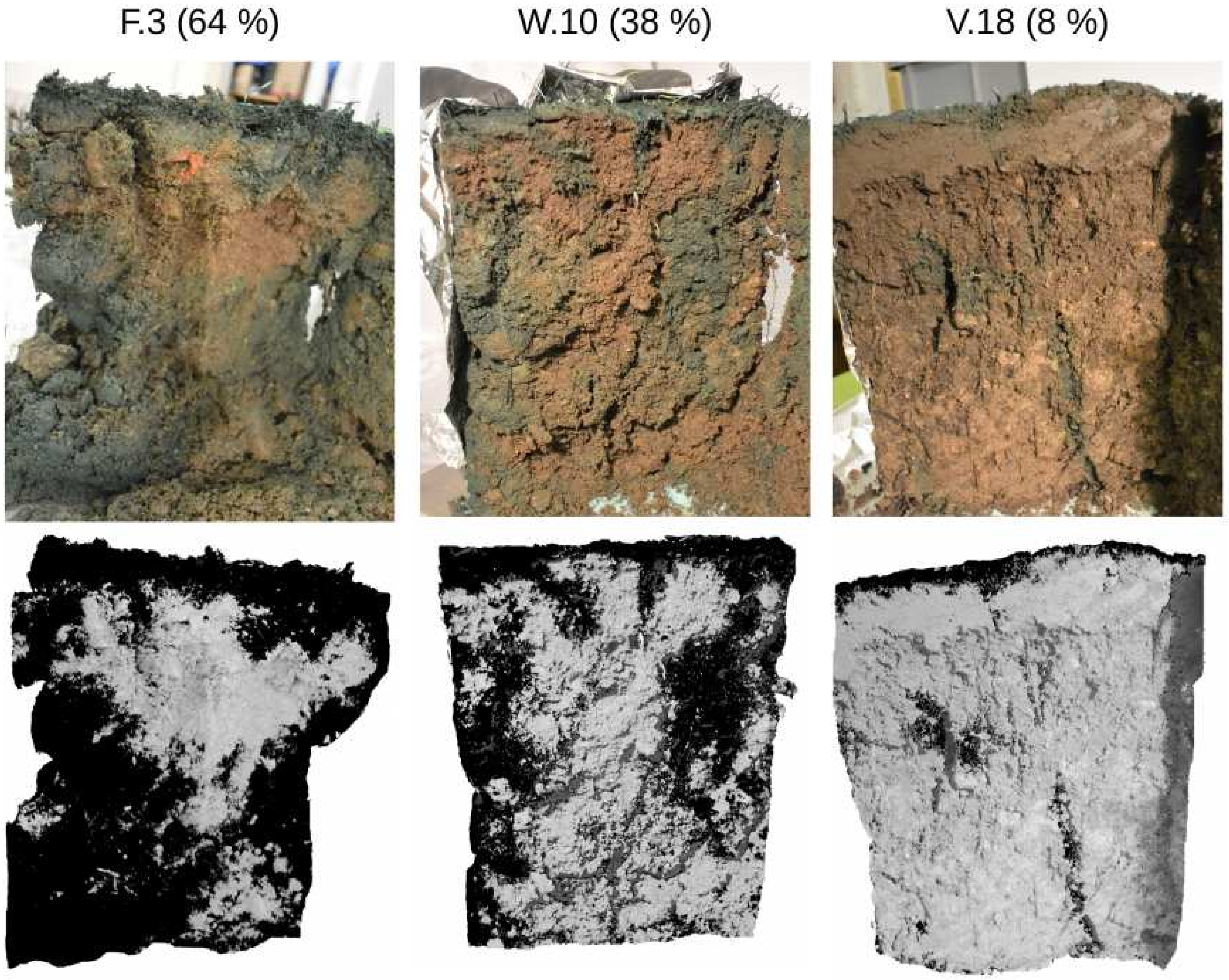

Figure S10. Brilliant blue stained soil columns (first row) and imageJ pictures (second row). The imageJ pictures show approximately the fraction of blue coloured area.

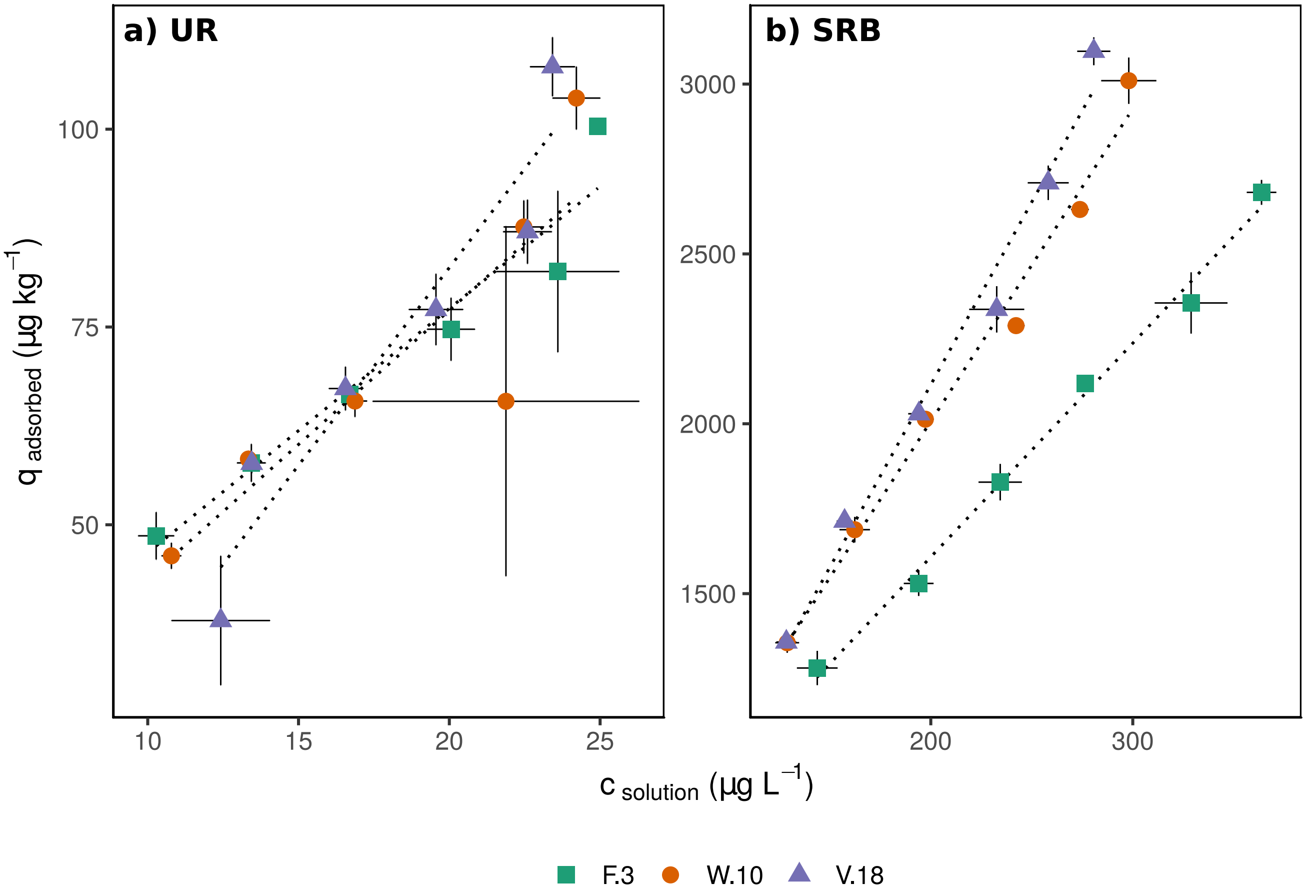

Figure S11. Adsorption isotherms for a) UR and b) SRB at the sites F.3, W.10 and V.18 determined in batch experiments. The errorbars in x- and y-direction represent the standard deviation of the tracer concentration measured in solution ($c_{solution}$) and the calculated content of adsorbed tracer ($q_{adsorbed}$) of three replicates each. Note the different concentration ranges of UR and SRB.

Tables

Table S1. K_d-values (linear sorption coefficients) for UR and SRB determined in batch experiments.

Column (site.age)	UR		SRB		UR	SRB
	k_d [a]	CI [b]	k_d [a]	CI [b]	r^2	
	$(L\,kg^{-1})$		$(L\,kg^{-1})$		(-)	
F.3	3.1 ± 0.4 **	[1.9, 4.2]	6.3 ± 0.3 ***	[5.6, 7.0]	0.93	0.99
W.10	3.4 ± 0.9 *	[0.9, 5.9]	9.2 ± 0.6 ***	[7.7, 10.7]	0.77	0.99
V.18	5.0 ± 0.8 **	[2.8, 7.2]	10.8 ± 0.7 ***	[8.8, 12.7]	0.91	0.98

[a]: Linear sorption coefficient K_d ± standard error.
[b]: 95 % confidence interval of linear sorption coefficients K_d.
Significance levels: (***): $p < 0.001$; (**): $p < 0.01$; (*): $p < 0.05$.

Table S2. Analytical parameter of the analysis of substances in LC-MS/MS. Fragmentation voltage (FV) and collision energy (CE) of the first and second transition.

Substance	Transition 1 (FV/CE)	Transition 2 (FV/CE)
Diuron	233.03 → 72.10 (96/17)	233.03 → 56.00 (96/57)
Terbutryn	242.15 → 186.10 (91/13)	242.15 → 68.00 (91/45)
OIT	214.13 → 102.00 (86/9)	214.13 → 57.10 (86/13)

Table S3. Total mean depths of soil cores. Additionally, the depths of the soil columns are listed.

Sample.type	F.3		W.10		V.18	
	Depth (cm)	n[a] (-)	Depth (cm)	n[a] (-)	Depth (cm)	n[a] (-)
soil core 1	16.7	4	23.8	5	29.0	6
soil core 2	18.5	4	25.0	5	29.0	6
soil core 3	15.5	4	25.0	5	28.0	6
soil core 4	23.6	5	25.0	5	25.0	5
Mean soil cores[b]	18.6 ± 3.6	4	24.7 ± 0.6	4	27.8 ± 1.9	4
Soil monoliths	21.0	1	25.0	1	25.0	1

[a] Number of single depth steps.
[b] Mean depths of soil 1 to 4 ± standard deviation.

Table S4. Repetitions of soil property analyses (according to available soil material). Depth classes: I: 0-5 cm, II: 5-10 cm, III: 10-15 cm, IV: 15-20 cm, V: 20-25 cm, VI: 25-30 cm

Depth.class	pH	OC	Stones	bd	Texture
			F.3		
I	12	4	4	4	4
II	12	4	4	4	-
III	12	4	4	4	4
IV	12	4	3	3	-
V	3	1	1	1	-
Sum	51	17	16	16	8
			W.10		
I	12	4	4	4	3
II	12	4	4	4	2
III	12	4	4	4	3
IV	12	4	4	4	1
V	12	4	4	4	1
Sum	60	20	20	20	10
			V.18		
I	12	4	4	4	1
II	12	4	4	4	2
III	12	4	4	4	2
IV	12	4	4	4	2
V	12	4	4	4	1
VI	9	3	2	2	-
Sum	69	23	22	22	8

Table S5. Target and actual initial concentrations for tracers and biocides. Actual initial concentrations for the tracers bromide, chloride, UR and SRB were calculated as the median from two replicates. Actual initial concentrations for the biocides diuron, terbutryn, OIT were calculated as the mean of three to four replicates.

Substance	Site	$c_{init,target}$ [a]	$c_{init,actual}$ [b] ($\mu g\,L^{-1}$)	c_{sd} [c]	No.Samples (-)
bromide	F.3	50000	61261	-	2
bromide	W.10	50000	61720	-	2
bromide	V.18	50000	60882	-	2
chloride	F.3	25000	25332	-	2
chloride	W.10	25000	25553	-	2
chloride	V.18	25000	25417	-	2
UR	F.3	10	8.9	-	2
UR	W.10	10	8.7	-	2
UR	V.18	10	9.6	-	2
SRB	F.3	400	399.0	-	2
SRB	W.10	400	409.4	-	2
SRB	V.18	400	400.0	-	2
diuron	F.3	50	46.7	2.0	3
diuron	W.10	50	53.9	5.2	3
diuron	V.18	50	49.0	5.8	4
terbutryn	F.3	50	43.2	1.5	4
terbutryn	W.10	50	46.1	0.5	4
terbutryn	V.18	50	44.6	0.5	3
OIT	F.3	50	48.4	5.4	3
OIT	W.10	50	55.7	0.7	3
OIT	V.18	50	58.3	6.3	4

[a] target initial concentration
[b] actual initial concentration measured in initial solutions
[c] standard deviation for initial biocide concentrations of n samples

Table S6. Depth-dependent soil properties (data to Fig. 1; means and standard diviations; repetitions are shown in Table S4).

Site.Age	Depth (-)	pH (% (w/w))	OC (% (w/w))	Stones ($g\,cm^{-3}$)	Bulk density
F.3	0-5	7.42 ± 0.09	1.43 ± 0.15	35.9 ± 1.9	1.54 ± 0.17
F.3	5-10	7.51 ± 0.07	1.07 ± 0.07	42.6 ± 5.9	1.51 ± 0.15
F.3	10-15	7.56 ± 0.06	0.95 ± 0.08	42.6 ± 9.0	1.58 ± 0.22
F.3	15-20	7.54 ± 0.09	0.99 ± 0.13	60.9 ± 22.9	1.56 ± 0.30
F.3	20-25	7.55 ± 0.00	1.17 ± -	43.4 ± -	1.6 ± -
W.10	0-5	7.09 ± 0.07	2.90 ± 0.24	16.9 ± 3.3	1.32 ± 0.06
W.10	5-10	7.22 ± 0.07	-	41.2 ± 4.5	1.52 ± 0.12
W.10	10-15	7.24 ± 0.07	1.33 ± 0.34	36.0 ± 3.0	1.48 ± 0.18
W.10	15-20	7.29 ± 0.04	1.15 ± 0.27	40.6 ± 4.8	1.39 ± 0.15
W.10	20-25	7.37 ± 0.07	0.98 ± 0.09	37.3 ± 4.1	1.48 ± 0.26
V.18	0-5	6.97 ± 0.05	3.65 ± 0.54	2.9 ± 2.3	1.02 ± 0.12
V.18	5-10	7.02 ± 0.06	3.01 ± 0.48	7.3 ± 5.3	1.12 ± 0.06
V.18	10-15	7.09 ± 0.04	1.88 ± 0.40	18.7 ± 2.5	1.31 ± 0.11
V.18	15-20	7.15 ± 0.06	1.23 ± 0.35	21.4 ± 1.3	1.50 ± 0.12
V.18	20-25	7.24 ± 0.05	0.85 ± 0.20	19.6 ± 3.8	1.50 ± 0.09
V.18	25-30	7.25 ± 0.03	0.76 ± 0.09	22.5 ± 1.9	1.50 ± 0.19

Table S7. Data of the soil column experiment (data to Fig. 2). Outflow: Amount of water at the bottom of the soil column at each time step; $c_{Outflow}$: concentration in the outflow at each time step; PV: pore volume.

Substance	Site.Age	Time (min)	Outflow (mL)	$c_{Outflow}$ (µg L^{-1})	PV (-)	$c_{Outflow}$ (%)
bromide	F.3	30	152	3260	0.1	5.3
bromide	F.3	60	298	3023	0.2	4.9
bromide	F.3	90	440	2862	0.3	4.7
bromide	F.3	120	570	3147	0.4	5.1
bromide	F.3	150	698	3985	0.5	6.5
bromide	F.3	180	821	6572	0.6	10.7
bromide	F.3	210	942	9095	0.7	14.8
bromide	F.3	240	1060	11613	0.8	19.0
bromide	F.3	270	1175	14711	0.9	24.0
bromide	F.3	300	1287	18013	1.0	29.4
bromide	F.3	330	1398	19697	1.1	32.2
bromide	F.3	360	1505	22900	1.2	37.4
bromide	F.3	390	1613	25312	1.3	41.3
bromide	F.3	420	1718	27013	1.3	44.1
bromide	F.3	450	1821	28675	1.4	46.8
bromide	F.3	480	1922	31118	1.5	50.8
bromide	F.3	510	2021	33145	1.6	54.1
bromide	F.3	540	2118	34771	1.6	56.8
bromide	F.3	570	2216	35631	1.7	58.2
bromide	F.3	600	2311	37709	1.8	61.6
bromide	F.3	640	2437	37946	1.9	61.9
bromide	F.3	680	2617	39668	2.0	64.8
bromide	F.3	720	2739	41631	2.1	68.0
bromide	F.3	760	2865	42046	2.2	68.6
bromide	F.3	800	2982	43230	2.3	70.6
bromide	F.3	840	3093	44298	2.4	72.3
bromide	F.3	920	3320	46705	2.6	76.2
bromide	F.3	1000	3546	47773	2.7	78.0
bromide	F.3	1040	3657	48848	2.8	79.7
bromide	F.3	1080	3767	52219	2.9	85.2
bromide	F.3	1160	3976	53832	3.1	87.9
bromide	F.3	1200	4081	51937	3.2	84.8
bromide	F.3	1240	4187	51317	3.2	83.8
bromide	F.3	1280	4292	52171	3.3	85.2
bromide	F.3	1380	4540	52719	3.5	86.1
bromide	F.3	1500	4825	55459	3.7	90.5
bromide	F.3	1560	4950	55334	3.8	90.3
bromide	F.3	1620	5060	55935	3.9	91.3
bromide	F.3	1680	5160	54365	4.0	88.7
bromide	W.10	15	204	3583	0.1	5.8
bromide	W.10	30	396	17949	0.2	29.1
bromide	W.10	45	584	32375	0.3	52.5
bromide	W.10	60	767	35350	0.4	57.3
bromide	W.10	75	948	37100	0.5	60.1
bromide	W.10	90	1125	42100	0.6	68.2
bromide	W.10	105	1301	41450	0.7	67.2
bromide	W.10	120	1473	42950	0.8	69.6
bromide	W.10	135	1645	46550	0.9	75.4
bromide	W.10	150	1814	45150	0.9	73.2
bromide	W.10	165	1981	45050	1.0	73.0

Substance	Site.Age	Time (min)	Outflow (mL)	$c_{Outflow}$ (µg L^{-1})	PV (-)	$c_{Outflow}$ (%)
bromide	W.10	180	2146	46150	1.1	74.8
bromide	W.10	195	2309	48350	1.2	78.3
bromide	W.10	210	2469	47600	1.3	77.1
bromide	W.10	225	2630	46650	1.4	75.6
bromide	W.10	240	2788	48000	1.5	77.8
bromide	W.10	255	2945	49750	1.5	80.6
bromide	W.10	270	3101	49850	1.6	80.8
bromide	W.10	285	3255	48800	1.7	79.1
bromide	W.10	300	3409	49750	1.8	80.6
bromide	W.10	315	3561	49550	1.9	80.3
bromide	W.10	330	3712	50100	1.9	81.2
bromide	W.10	345	3857	51250	2.0	83.0
bromide	W.10	360	4006	52750	2.1	85.5
bromide	W.10	375	4154	51750	2.2	83.8
bromide	W.10	390	4300	52150	2.3	84.5
bromide	W.10	420	4589	52500	2.4	85.1
bromide	W.10	435	4733	53600	2.5	86.8
bromide	W.10	450	4878	53250	2.6	86.3
bromide	W.10	465	5016	54250	2.6	87.9
bromide	W.10	480	5159	53750	2.7	87.1
bromide	W.10	495	5301	54100	2.8	87.7
bromide	W.10	510	5436	55800	2.8	90.4
bromide	W.10	525	5574	53350	2.9	86.4
bromide	W.10	540	5710	52950	3.0	85.8
bromide	W.10	555	5844	55400	3.1	89.8
bromide	W.10	570	5978	54350	3.1	88.1
bromide	W.10	585	6112	53450	3.2	86.6
bromide	W.10	600	6244	55200	3.3	89.4
bromide	W.10	620	6419	54650	3.4	88.5
bromide	W.10	640	6592	53100	3.5	86.0
bromide	W.10	660	6764	53800	3.5	87.2
bromide	W.10	680	6934	56750	3.6	91.9
bromide	W.10	700	7101	55750	3.7	90.3
bromide	W.10	720	7269	55200	3.8	89.4
bromide	W.10	760	7599	57550	4.0	93.2
bromide	V.18	15	290	9664	0.1	15.9
bromide	V.18	30	562	40760	0.2	66.9
bromide	V.18	45	827	46480	0.3	76.3
bromide	V.18	60	1111	50311	0.4	82.6
bromide	V.18	75	1367	55396	0.5	91.0
bromide	V.18	90	1621	54357	0.6	89.3
bromide	V.18	105	1872	58116	0.7	95.5
bromide	V.18	120	2120	55661	0.8	91.4
bromide	V.18	135	2366	56766	0.8	93.2
bromide	V.18	150	2611	56665	0.9	93.1
bromide	V.18	165	2852	56909	1.0	93.5
bromide	V.18	180	3093	58243	1.1	95.7
bromide	V.18	195	3331	57074	1.2	93.7
bromide	V.18	210	3568	60791	1.3	99.8
bromide	V.18	225	3802	57871	1.4	95.1
bromide	V.18	240	4035	58522	1.4	96.1
bromide	V.18	255	4268	59561	1.5	97.8
bromide	V.18	270	4500	58564	1.6	96.2

Substance	Site.Age	Time (min)	Outflow (mL)	$c_{Outflow}$ ($\mu g\,L^{-1}$)	PV (-)	$c_{Outflow}$ (%)
bromide	V.18	285	4729	57974	1.7	95.2
bromide	V.18	300	4958	59832	1.8	98.3
bromide	V.18	315	5184	59153	1.9	97.2
bromide	V.18	330	5409	60511	1.9	99.4
bromide	V.18	345	5632	59187	2.0	97.2
bromide	V.18	360	5856	59752	2.1	98.1
bromide	V.18	375	6077	59018	2.2	96.9
bromide	V.18	390	6296	60118	2.2	98.7
bromide	V.18	420	6727	59972	2.4	98.5
bromide	V.18	435	6942	59454	2.5	97.7
bromide	V.18	450	7155	59774	2.6	98.2
bromide	V.18	465	7362	59663	2.6	98.0
bromide	V.18	480	7571	59748	2.7	98.1
bromide	V.18	495	7779	60128	2.8	98.8
bromide	V.18	510	7984	62019	2.9	101.9
bromide	V.18	525	8188	59459	2.9	97.7
bromide	V.18	540	8392	62283	3.0	102.3
bromide	V.18	555	8594	59741	3.1	98.1
bromide	V.18	570	8794	59785	3.1	98.2
bromide	V.18	585	8992	60527	3.2	99.4
bromide	V.18	600	9187	61922	3.3	101.7
bromide	V.18	620	9451	60858	3.4	100.0
bromide	V.18	640	9707	61205	3.5	100.5
bromide	V.18	660	9964	63916	3.6	105.0
bromide	V.18	680	10220	60666	3.6	99.6
bromide	V.18	700	10471	60349	3.7	99.1
bromide	V.18	720	10723	62429	3.8	102.5
bromide	V.18	740	10978	63316	3.9	104.0
chloride	F.3	30	152	1646	0.1	6.5
chloride	F.3	60	298	1826	0.2	7.2
chloride	F.3	90	440	1710	0.3	6.7
chloride	F.3	120	570	1835	0.4	7.2
chloride	F.3	150	698	3480	0.5	13.7
chloride	F.3	180	821	3318	0.6	13.1
chloride	F.3	210	942	4526	0.7	17.9
chloride	F.3	240	1060	5712	0.8	22.5
chloride	F.3	270	1175	8040	0.9	31.7
chloride	F.3	300	1287	8019	1.0	31.7
chloride	F.3	330	1398	9392	1.1	37.1
chloride	F.3	360	1505	10460	1.2	41.3
chloride	F.3	390	1613	11114	1.3	43.9
chloride	F.3	420	1718	11935	1.3	47.1
chloride	F.3	450	1821	12604	1.4	49.8
chloride	F.3	480	1922	13828	1.5	54.6
chloride	F.3	510	2021	14479	1.6	57.2
chloride	F.3	540	2118	15119	1.6	59.7
chloride	F.3	570	2216	15497	1.7	61.2
chloride	F.3	600	2311	16627	1.8	65.6
chloride	F.3	640	2437	16573	1.9	65.4
chloride	F.3	680	2617	17179	2.0	67.8
chloride	F.3	720	2739	17762	2.1	70.1
chloride	F.3	760	2865	18620	2.2	73.5
chloride	F.3	800	2982	18698	2.3	73.8

Substance	Site.Age	Time (min)	Outflow (mL)	$c_{Outflow}$ (µg L^{-1})	PV (-)	$c_{Outflow}$ (%)
chloride	F.3	840	3093	19522	2.4	77.1
chloride	F.3	920	3320	19854	2.6	78.4
chloride	F.3	1000	3546	20569	2.7	81.2
chloride	F.3	1040	3657	20871	2.8	82.4
chloride	F.3	1080	3767	21286	2.9	84.0
chloride	F.3	1160	3976	21508	3.1	84.9
chloride	F.3	1200	4081	22017	3.2	86.9
chloride	F.3	1240	4187	21969	3.2	86.7
chloride	F.3	1280	4292	22484	3.3	88.8
chloride	F.3	1380	4540	22453	3.5	88.6
chloride	F.3	1500	4825	23151	3.7	91.4
chloride	F.3	1560	4950	24538	3.8	96.9
chloride	F.3	1620	5060	23602	3.9	93.2
chloride	F.3	1680	5160	23929	4.0	94.5
chloride	W.10	15	204	1567	0.1	6.1
chloride	W.10	30	396	7624	0.2	29.8
chloride	W.10	45	584	11754	0.3	46.0
chloride	W.10	60	767	13506	0.4	52.9
chloride	W.10	75	948	15650	0.5	61.2
chloride	W.10	90	1125	17700	0.6	69.3
chloride	W.10	105	1301	17500	0.7	68.5
chloride	W.10	120	1473	17600	0.8	68.9
chloride	W.10	135	1645	19250	0.9	75.3
chloride	W.10	150	1814	19100	0.9	74.7
chloride	W.10	165	1981	19450	1.0	76.1
chloride	W.10	180	2146	19050	1.1	74.5
chloride	W.10	195	2309	20400	1.2	79.8
chloride	W.10	210	2469	21000	1.3	82.2
chloride	W.10	225	2630	19800	1.4	77.5
chloride	W.10	240	2788	20000	1.5	78.3
chloride	W.10	255	2945	20600	1.5	80.6
chloride	W.10	270	3101	20900	1.6	81.8
chloride	W.10	285	3255	21300	1.7	83.4
chloride	W.10	300	3409	22250	1.8	87.1
chloride	W.10	315	3561	20700	1.9	81.0
chloride	W.10	330	3712	20650	1.9	80.8
chloride	W.10	345	3857	22300	2.0	87.3
chloride	W.10	360	4006	21050	2.1	82.4
chloride	W.10	375	4154	21200	2.2	83.0
chloride	W.10	390	4300	22100	2.3	86.5
chloride	W.10	420	4589	21650	2.4	84.7
chloride	W.10	435	4733	21550	2.5	84.3
chloride	W.10	450	4878	22100	2.6	86.5
chloride	W.10	465	5016	21900	2.6	85.7
chloride	W.10	480	5159	22050	2.7	86.3
chloride	W.10	495	5301	21950	2.8	85.9
chloride	W.10	510	5436	22150	2.8	86.7
chloride	W.10	525	5574	22150	2.9	86.7
chloride	W.10	540	5710	22000	3.0	86.1
chloride	W.10	555	5844	22400	3.1	87.7
chloride	W.10	570	5978	22350	3.1	87.5
chloride	W.10	585	6112	22700	3.2	88.8
chloride	W.10	600	6244	23000	3.3	90.0

Substance	Site.Age	Time (min)	Outflow (mL)	$c_{Outflow}$ (µg L^{-1})	PV (-)	$c_{Outflow}$ (%)
chloride	W.10	620	6419	23200	3.4	90.8
chloride	W.10	640	6592	23800	3.5	93.1
chloride	W.10	660	6764	22650	3.5	88.6
chloride	W.10	680	6934	22400	3.6	87.7
chloride	W.10	700	7101	22350	3.7	87.5
chloride	W.10	720	7269	22550	3.8	88.2
chloride	W.10	760	7599	23650	4.0	92.6
chloride	V.18	15	290	4670	0.1	18.4
chloride	V.18	30	562	16162	0.2	63.6
chloride	V.18	45	827	19117	0.3	75.2
chloride	V.18	60	1111	20611	0.4	81.1
chloride	V.18	75	1367	21691	0.5	85.3
chloride	V.18	90	1621	22385	0.6	88.1
chloride	V.18	105	1872	22876	0.7	90.0
chloride	V.18	120	2120	23318	0.8	91.7
chloride	V.18	135	2366	23318	0.8	91.7
chloride	V.18	150	2611	23750	0.9	93.4
chloride	V.18	165	2852	23547	1.0	92.6
chloride	V.18	180	3093	23836	1.1	93.8
chloride	V.18	195	3331	24573	1.2	96.7
chloride	V.18	210	3568	24012	1.3	94.5
chloride	V.18	225	3802	23881	1.4	94.0
chloride	V.18	240	4035	23832	1.4	93.8
chloride	V.18	255	4268	24672	1.5	97.1
chloride	V.18	270	4500	23977	1.6	94.3
chloride	V.18	285	4729	24143	1.7	95.0
chloride	V.18	300	4958	24067	1.8	94.7
chloride	V.18	315	5184	24525	1.9	96.5
chloride	V.18	330	5409	24656	1.9	97.0
chloride	V.18	345	5632	24779	2.0	97.5
chloride	V.18	360	5856	24235	2.1	95.3
chloride	V.18	375	6077	24312	2.2	95.7
chloride	V.18	390	6296	24536	2.2	96.5
chloride	V.18	420	6727	24626	2.4	96.9
chloride	V.18	435	6942	24544	2.5	96.6
chloride	V.18	450	7155	24434	2.6	96.1
chloride	V.18	465	7362	24399	2.6	96.0
chloride	V.18	480	7571	25066	2.7	98.6
chloride	V.18	495	7779	24547	2.8	96.6
chloride	V.18	510	7984	24557	2.9	96.6
chloride	V.18	525	8188	24558	2.9	96.6
chloride	V.18	540	8392	24787	3.0	97.5
chloride	V.18	555	8594	24657	3.1	97.0
chloride	V.18	570	8794	24558	3.1	96.6
chloride	V.18	585	8992	26045	3.2	102.5
chloride	V.18	600	9187	25013	3.3	98.4
chloride	V.18	620	9451	24774	3.4	97.5
chloride	V.18	640	9707	24629	3.5	96.9
chloride	V.18	660	9964	26064	3.6	102.5
chloride	V.18	680	10220	25038	3.6	98.5
chloride	V.18	700	10471	24749	3.7	97.4
chloride	V.18	720	10723	25006	3.8	98.4
chloride	V.18	740	10978	24766	3.9	97.4

Substance	Site.Age	Time (min)	Outflow (mL)	$c_{Outflow}$ (µg L^{-1})	PV (-)	$c_{Outflow}$ (%)
SRB	F.3	30	152	0.9	0.1	0.2
SRB	F.3	60	298	0.0	0.2	0.0
SRB	F.3	90	440	0.0	0.3	0.0
SRB	F.3	120	570	0.1	0.4	0.0
SRB	F.3	150	698	0.4	0.5	0.1
SRB	F.3	180	821	1.8	0.6	0.5
SRB	F.3	210	942	4.7	0.7	1.2
SRB	F.3	240	1060	8.4	0.8	2.1
SRB	F.3	270	1175	13.4	0.9	3.4
SRB	F.3	300	1287	18.7	1.0	4.7
SRB	F.3	330	1398	22.8	1.1	5.7
SRB	F.3	360	1505	29.4	1.2	7.4
SRB	F.3	390	1613	33.8	1.3	8.5
SRB	F.3	420	1718	38.4	1.3	9.6
SRB	F.3	450	1821	42.3	1.4	10.6
SRB	F.3	480	1922	46.1	1.5	11.6
SRB	F.3	510	2021	49.1	1.6	12.3
SRB	F.3	540	2118	53.2	1.6	13.3
SRB	F.3	570	2216	57.4	1.7	14.4
SRB	F.3	600	2311	59.5	1.8	14.9
SRB	F.3	640	2437	63.5	1.9	15.9
SRB	F.3	680	2617	66.0	2.0	16.5
SRB	F.3	720	2739	70.9	2.1	17.8
SRB	F.3	760	2865	72.7	2.2	18.2
SRB	F.3	800	2982	75.0	2.3	18.8
SRB	F.3	840	3093	76.5	2.4	19.2
SRB	F.3	920	3320	82.4	2.6	20.6
SRB	F.3	960	3434	85.3	2.7	21.4
SRB	F.3	1000	3546	88.8	2.7	22.3
SRB	F.3	1040	3657	89.9	2.8	22.5
SRB	F.3	1080	3767	91.9	2.9	23.0
SRB	F.3	1120	3870	91.9	3.0	23.0
SRB	F.3	1160	3976	93.1	3.1	23.3
SRB	F.3	1200	4081	93.4	3.2	23.4
SRB	F.3	1240	4187	95.0	3.2	23.8
SRB	F.3	1280	4292	95.5	3.3	23.9
SRB	F.3	1320	4392	97.1	3.4	24.3
SRB	F.3	1380	4540	98.4	3.5	24.7
SRB	F.3	1440	4684	100.8	3.6	25.3
SRB	F.3	1500	4825	101.0	3.7	25.3
SRB	F.3	1560	4950	102.9	3.8	25.8
SRB	F.3	1620	5060	103.9	3.9	26.0
SRB	F.3	1680	5160	103.8	4.0	26.0
SRB	W.10	15	204	7.5	0.1	1.8
SRB	W.10	30	396	82.3	0.2	20.1
SRB	W.10	45	584	162.2	0.3	39.6
SRB	W.10	60	767	199.3	0.4	48.7
SRB	W.10	75	948	229.8	0.5	56.1
SRB	W.10	90	1125	237.3	0.6	58.0
SRB	W.10	105	1301	248.1	0.7	60.6
SRB	W.10	120	1473	257.7	0.8	63.0
SRB	W.10	135	1645	260.6	0.9	63.6
SRB	W.10	150	1814	266.1	0.9	65.0

Substance	Site.Age	Time (min)	Outflow (mL)	$c_{Outflow}$ ($\mu g\,L^{-1}$)	PV (-)	$c_{Outflow}$ (%)
SRB	W.10	165	1981	267.2	1.0	65.3
SRB	W.10	180	2146	269.3	1.1	65.8
SRB	W.10	195	2309	272.9	1.2	66.7
SRB	W.10	210	2469	274.7	1.3	67.1
SRB	W.10	225	2630	276.1	1.4	67.4
SRB	W.10	240	2788	277.7	1.5	67.8
SRB	W.10	255	2945	278.0	1.5	67.9
SRB	W.10	270	3101	281.4	1.6	68.7
SRB	W.10	285	3255	279.9	1.7	68.4
SRB	W.10	300	3409	282.0	1.8	68.9
SRB	W.10	315	3561	288.3	1.9	70.4
SRB	W.10	330	3712	279.8	1.9	68.3
SRB	W.10	345	3857	283.8	2.0	69.3
SRB	W.10	360	4006	283.9	2.1	69.3
SRB	W.10	375	4154	285.4	2.2	69.7
SRB	W.10	390	4300	285.7	2.3	69.8
SRB	W.10	420	4589	284.7	2.4	69.5
SRB	W.10	435	4733	290.8	2.5	71.0
SRB	W.10	450	4878	285.8	2.6	69.8
SRB	W.10	465	5016	286.0	2.6	69.8
SRB	W.10	480	5159	282.4	2.7	69.0
SRB	W.10	495	5301	289.5	2.8	70.7
SRB	W.10	510	5436	288.8	2.8	70.5
SRB	W.10	525	5574	289.3	2.9	70.7
SRB	W.10	540	5710	285.5	3.0	69.7
SRB	W.10	555	5844	289.7	3.1	70.8
SRB	W.10	585	6112	287.0	3.2	70.1
SRB	W.10	600	6244	290.1	3.3	70.9
SRB	W.10	620	6419	294.6	3.4	72.0
SRB	W.10	640	6592	286.7	3.5	70.0
SRB	W.10	660	6764	281.3	3.5	68.7
SRB	W.10	680	6934	286.7	3.6	70.0
SRB	W.10	700	7101	287.5	3.7	70.2
SRB	W.10	720	7269	284.8	3.8	69.6
SRB	W.10	740	7434	284.3	3.9	69.4
SRB	W.10	760	7599	290.2	4.0	70.9
SRB	V.18	15	290	39.8	0.1	10.0
SRB	V.18	30	562	214.7	0.2	53.7
SRB	V.18	45	827	276.4	0.3	69.1
SRB	V.18	60	1111	301.7	0.4	75.4
SRB	V.18	75	1367	324.4	0.5	81.1
SRB	V.18	90	1621	337.2	0.6	84.3
SRB	V.18	105	1872	340.2	0.7	85.0
SRB	V.18	120	2120	343.7	0.8	85.9
SRB	V.18	135	2366	346.4	0.8	86.6
SRB	V.18	150	2611	349.3	0.9	87.3
SRB	V.18	165	2852	359.2	1.0	89.8
SRB	V.18	180	3093	349.9	1.1	87.5
SRB	V.18	195	3331	355.8	1.2	88.9
SRB	V.18	210	3568	356.7	1.3	89.2
SRB	V.18	225	3802	355.2	1.4	88.8
SRB	V.18	240	4035	358.7	1.4	89.7
SRB	V.18	255	4268	357.8	1.5	89.4

Substance	Site.Age	Time (min)	Outflow (mL)	$c_{Outflow}$ (µg L^{-1})	PV (-)	$c_{Outflow}$ (%)
SRB	V.18	270	4500	361.1	1.6	90.3
SRB	V.18	285	4729	360.4	1.7	90.1
SRB	V.18	300	4958	363.4	1.8	90.8
SRB	V.18	315	5184	358.2	1.9	89.5
SRB	V.18	330	5409	360.1	1.9	90.0
SRB	V.18	345	5632	360.2	2.0	90.0
SRB	V.18	360	5856	362.3	2.1	90.6
SRB	V.18	375	6077	359.2	2.2	89.8
SRB	V.18	390	6296	358.1	2.2	89.5
SRB	V.18	420	6727	355.1	2.4	88.8
SRB	V.18	435	6942	358.5	2.5	89.6
SRB	V.18	450	7155	367.4	2.6	91.8
SRB	V.18	465	7362	357.2	2.6	89.3
SRB	V.18	480	7571	360.8	2.7	90.2
SRB	V.18	495	7779	359.1	2.8	89.8
SRB	V.18	510	7984	370.2	2.9	92.5
SRB	V.18	525	8188	365.1	2.9	91.3
SRB	V.18	540	8392	366.3	3.0	91.6
SRB	V.18	555	8594	354.6	3.1	88.6
SRB	V.18	570	8794	365.6	3.1	91.4
SRB	V.18	585	8992	361.4	3.2	90.3
SRB	V.18	600	9187	358.4	3.3	89.6
SRB	V.18	620	9451	364.8	3.4	91.2
SRB	V.18	640	9707	359.5	3.5	89.9
SRB	V.18	660	9964	361.3	3.6	90.3
SRB	V.18	680	10220	358.7	3.6	89.7
SRB	V.18	700	10471	364.7	3.7	91.2
SRB	V.18	720	10723	345.5	3.8	86.4
SRB	V.18	740	10978	349.5	3.9	87.4
UR	F.3	30	152	0.7	0.1	8.1
UR	F.3	60	298	0.6	0.2	6.8
UR	F.3	90	440	0.7	0.3	7.7
UR	F.3	120	570	0.7	0.4	8.1
UR	F.3	150	698	0.7	0.5	8.1
UR	F.3	180	821	0.8	0.6	9.2
UR	F.3	210	942	1.0	0.7	10.9
UR	F.3	240	1060	1.2	0.8	13.5
UR	F.3	270	1175	1.5	0.9	16.7
UR	F.3	300	1287	1.7	1.0	19.4
UR	F.3	330	1398	2.0	1.1	22.2
UR	F.3	360	1505	2.2	1.2	24.6
UR	F.3	390	1613	2.4	1.3	26.7
UR	F.3	420	1718	2.5	1.3	28.4
UR	F.3	450	1821	2.7	1.4	30.1
UR	F.3	480	1922	2.8	1.5	31.8
UR	F.3	510	2021	2.9	1.6	33.2
UR	F.3	540	2118	3.1	1.6	34.4
UR	F.3	570	2216	3.2	1.7	35.7
UR	F.3	600	2311	3.3	1.8	36.8
UR	F.3	640	2437	3.3	1.9	37.4
UR	F.3	680	2617	3.5	2.0	39.0
UR	F.3	720	2739	3.6	2.1	40.6
UR	F.3	760	2865	3.7	2.2	41.6

Substance	Site.Age	Time (min)	Outflow (mL)	$c_{Outflow}$ (µg L^{-1})	PV (-)	$c_{Outflow}$ (%)
UR	F.3	800	2982	3.8	2.3	42.5
UR	F.3	840	3093	3.8	2.4	43.1
UR	F.3	920	3320	4.0	2.6	45.6
UR	F.3	960	3434	4.1	2.7	45.8
UR	F.3	1000	3546	4.2	2.7	47.3
UR	F.3	1040	3657	4.2	2.8	47.6
UR	F.3	1080	3767	4.2	2.9	47.8
UR	F.3	1120	3870	4.2	3.0	47.8
UR	F.3	1160	3976	4.3	3.1	48.3
UR	F.3	1200	4081	4.3	3.2	48.8
UR	F.3	1240	4187	4.3	3.2	49.0
UR	F.3	1280	4292	4.4	3.3	49.9
UR	F.3	1320	4392	4.4	3.4	50.2
UR	F.3	1380	4540	4.5	3.5	50.6
UR	F.3	1440	4684	4.6	3.6	51.5
UR	F.3	1500	4825	4.6	3.7	51.6
UR	F.3	1560	4950	4.6	3.8	52.0
UR	F.3	1620	5060	4.7	3.9	52.8
UR	F.3	1680	5160	4.7	4.0	52.5
UR	W.10	15	204	0.5	0.1	5.9
UR	W.10	30	396	2.7	0.2	31.2
UR	W.10	45	584	4.3	0.3	50.1
UR	W.10	60	767	5.0	0.4	58.0
UR	W.10	75	948	5.3	0.5	61.3
UR	W.10	90	1125	5.5	0.6	63.5
UR	W.10	105	1301	5.9	0.7	68.2
UR	W.10	120	1473	6.1	0.8	69.9
UR	W.10	135	1645	5.9	0.9	68.4
UR	W.10	150	1814	6.2	0.9	71.4
UR	W.10	165	1981	6.3	1.0	72.2
UR	W.10	180	2146	6.3	1.1	72.8
UR	W.10	195	2309	6.3	1.2	73.1
UR	W.10	210	2469	6.4	1.3	74.0
UR	W.10	225	2630	6.5	1.4	74.7
UR	W.10	240	2788	6.4	1.5	74.1
UR	W.10	255	2945	6.6	1.5	76.2
UR	W.10	270	3101	6.5	1.6	74.4
UR	W.10	285	3255	6.6	1.7	75.6
UR	W.10	300	3409	6.7	1.8	76.9
UR	W.10	315	3561	6.8	1.9	78.2
UR	W.10	330	3712	6.7	1.9	77.3
UR	W.10	345	3857	6.7	2.0	77.3
UR	W.10	360	4006	6.7	2.1	77.0
UR	W.10	375	4154	6.8	2.2	78.5
UR	W.10	390	4300	6.7	2.3	77.6
UR	W.10	420	4589	6.8	2.4	78.8
UR	W.10	435	4733	6.8	2.5	78.8
UR	W.10	450	4878	6.8	2.6	78.0
UR	W.10	465	5016	6.8	2.6	78.4
UR	W.10	480	5159	6.8	2.7	78.6
UR	W.10	495	5301	6.9	2.8	79.4
UR	W.10	510	5436	6.9	2.8	79.7
UR	W.10	525	5574	6.8	2.9	78.6

Substance	Site.Age	Time (min)	Outflow (mL)	$c_{Outflow}$ ($\mu g\,L^{-1}$)	PV (-)	$c_{Outflow}$ (%)
UR	W.10	540	5710	7.1	3.0	81.6
UR	W.10	555	5844	7.0	3.1	80.2
UR	W.10	585	6112	7.1	3.2	82.1
UR	W.10	600	6244	6.9	3.3	79.3
UR	W.10	620	6419	6.8	3.4	77.9
UR	W.10	640	6592	6.9	3.5	79.1
UR	W.10	660	6764	6.8	3.5	78.1
UR	W.10	680	6934	6.9	3.6	79.0
UR	W.10	700	7101	7.0	3.7	80.4
UR	W.10	720	7269	6.9	3.8	79.1
UR	W.10	740	7434	6.8	3.9	77.9
UR	W.10	760	7599	6.8	4.0	78.8
UR	V.18	15	290	1.7	0.1	17.6
UR	V.18	30	562	6.5	0.2	68.1
UR	V.18	45	827	7.7	0.3	80.8
UR	V.18	60	1111	8.4	0.4	87.4
UR	V.18	75	1367	8.8	0.5	92.0
UR	V.18	90	1621	9.1	0.6	94.6
UR	V.18	105	1872	9.1	0.7	95.1
UR	V.18	120	2120	9.2	0.8	95.7
UR	V.18	135	2366	9.3	0.8	96.9
UR	V.18	150	2611	9.3	0.9	96.5
UR	V.18	165	2852	9.5	1.0	98.8
UR	V.18	180	3093	9.4	1.1	98.1
UR	V.18	195	3331	9.5	1.2	98.6
UR	V.18	210	3568	9.5	1.3	99.3
UR	V.18	225	3802	9.5	1.4	99.1
UR	V.18	240	4035	9.5	1.4	99.5
UR	V.18	255	4268	9.6	1.5	100.2
UR	V.18	270	4500	9.5	1.6	98.9
UR	V.18	285	4729	9.5	1.7	99.3
UR	V.18	300	4958	9.5	1.8	99.5
UR	V.18	315	5184	9.3	1.9	97.3
UR	V.18	330	5409	9.4	1.9	97.9
UR	V.18	345	5632	9.4	2.0	97.9
UR	V.18	360	5856	9.5	2.1	98.9
UR	V.18	375	6077	9.5	2.2	99.2
UR	V.18	390	6296	9.5	2.2	99.1
UR	V.18	420	6727	9.4	2.4	98.3
UR	V.18	435	6942	9.6	2.5	99.8
UR	V.18	450	7155	9.6	2.6	100.2
UR	V.18	465	7362	9.5	2.6	98.9
UR	V.18	480	7571	9.6	2.7	100.3
UR	V.18	495	7779	9.5	2.8	99.4
UR	V.18	510	7984	9.6	2.9	100.0
UR	V.18	525	8188	9.5	2.9	99.2
UR	V.18	540	8392	9.6	3.0	99.7
UR	V.18	555	8594	9.5	3.1	99.4
UR	V.18	570	8794	9.6	3.1	99.8
UR	V.18	585	8992	9.6	3.2	99.7
UR	V.18	600	9187	9.4	3.3	98.1
UR	V.18	620	9451	9.6	3.4	99.9
UR	V.18	640	9707	9.5	3.5	99.6

Substance	Site.Age	Time (min)	Outflow (mL)	$c_{Outflow}$ ($\mu g\,L^{-1}$)	PV (-)	$c_{Outflow}$ (%)
UR	V.18	660	9964	9.5	3.6	99.2
UR	V.18	680	10220	9.5	3.6	99.1
UR	V.18	700	10471	9.5	3.7	99.2
UR	V.18	720	10723	9.2	3.8	96.4
UR	V.18	740	10978	9.3	3.9	96.8
diuron	F.3	60	298	0.0	0.2	0.0
diuron	F.3	150	698	0.4	0.5	0.8
diuron	F.3	240	1060	0.0	0.8	0.0
diuron	F.3	360	1505	0.7	1.2	1.6
diuron	F.3	480	1922	0.4	1.5	0.8
diuron	F.3	640	2437	2.3	1.9	5.0
diuron	F.3	840	3093	2.7	2.4	5.8
diuron	F.3	960	3434	3.3	2.7	7.1
diuron	F.3	1120	3870	4.5	3.0	9.7
diuron	F.3	1440	4684	2.9	3.6	6.2
diuron	W.10	30	396	1.9	0.2	3.4
diuron	W.10	75	948	17.0	0.5	31.5
diuron	W.10	120	1473	18.0	0.8	33.4
diuron	W.10	180	2146	35.8	1.1	66.5
diuron	W.10	240	2788	39.0	1.5	72.5
diuron	W.10	315	3561	38.2	1.9	71.0
diuron	W.10	390	4300	22.0	2.3	40.8
diuron	W.10	495	5301	20.6	2.8	38.3
diuron	W.10	600	6244	38.7	3.3	71.8
diuron	W.10	760	7599	40.7	4.0	75.6
diuron	V.18	30	562	10.8	0.2	22.0
diuron	V.18	75	1367	39.6	0.5	80.7
diuron	V.18	120	2120	45.6	0.8	92.9
diuron	V.18	180	3093	52.2	1.1	106.4
diuron	V.18	240	4035	52.0	1.4	106.0
diuron	V.18	315	5184	53.2	1.9	108.5
diuron	V.18	390	6296	59.3	2.2	121.0
diuron	V.18	495	7779	54.2	2.8	110.5
diuron	V.18	600	9187	47.3	3.3	96.4
octhilinone	F.3	60	298	0.0	0.2	0.0
octhilinone	F.3	150	698	0.0	0.5	0.0
octhilinone	F.3	240	1060	0.0	0.8	0.0
octhilinone	F.3	360	1505	0.0	1.2	0.0
octhilinone	F.3	480	1922	0.0	1.5	0.0
octhilinone	F.3	640	2437	0.0	1.9	0.0
octhilinone	F.3	840	3093	0.0	2.4	0.0
octhilinone	F.3	960	3434	0.0	2.7	0.0
octhilinone	F.3	1120	3870	0.0	3.0	0.0
octhilinone	F.3	1440	4684	0.0	3.6	0.0
octhilinone	W.10	30	396	2.7	0.2	4.9
octhilinone	W.10	75	948	10.1	0.5	18.1
octhilinone	W.10	120	1473	13.6	0.8	24.4
octhilinone	W.10	180	2146	12.9	1.1	23.2
octhilinone	W.10	240	2788	13.2	1.5	23.7
octhilinone	W.10	315	3561	13.0	1.9	23.3
octhilinone	W.10	390	4300	6.4	2.3	11.6
octhilinone	W.10	495	5301	8.6	2.8	15.4
octhilinone	W.10	600	6244	4.7	3.3	8.5

Substance	Site.Age	Time (min)	Outflow (mL)	$c_{Outflow}$ (µg L^{-1})	PV (-)	$c_{Outflow}$ (%)
octhilinone	W.10	760	7599	7.9	4.0	14.3
octhilinone	V.18	30	562	13.7	0.2	23.4
octhilinone	V.18	75	1367	11.9	0.5	20.4
octhilinone	V.18	120	2120	8.2	0.8	14.1
octhilinone	V.18	180	3093	12.1	1.1	20.8
octhilinone	V.18	240	4035	18.2	1.4	31.2
octhilinone	V.18	315	5184	14.5	1.9	24.8
octhilinone	V.18	390	6296	17.0	2.2	29.1
octhilinone	V.18	495	7779	7.6	2.8	13.1
octhilinone	V.18	600	9187	7.9	3.3	13.6
terbutryn	F.3	60	298	0.0	0.2	0.0
terbutryn	F.3	150	698	0.0	0.5	0.0
terbutryn	F.3	240	1060	0.7	0.8	1.5
terbutryn	F.3	360	1505	0.7	1.2	1.7
terbutryn	F.3	480	1922	1.0	1.5	2.2
terbutryn	F.3	640	2437	1.4	1.9	3.2
terbutryn	F.3	840	3093	1.2	2.4	2.7
terbutryn	F.3	960	3434	1.5	2.7	3.5
terbutryn	F.3	1120	3870	2.0	3.0	4.7
terbutryn	F.3	1440	4684	2.5	3.6	5.9
terbutryn	W.10	30	396	1.5	0.2	3.2
terbutryn	W.10	75	948	12.6	0.5	27.3
terbutryn	W.10	120	1473	18.9	0.8	41.0
terbutryn	W.10	180	2146	19.6	1.1	42.6
terbutryn	W.10	240	2788	24.8	1.5	53.9
terbutryn	W.10	315	3561	25.6	1.9	55.5
terbutryn	W.10	390	4300	22.6	2.3	48.9
terbutryn	W.10	495	5301	14.8	2.8	32.2
terbutryn	W.10	600	6244	15.6	3.3	33.9
terbutryn	W.10	760	7599	21.2	4.0	46.0
terbutryn	V.18	30	562	5.7	0.2	12.7
terbutryn	V.18	75	1367	11.5	0.5	25.8
terbutryn	V.18	120	2120	13.6	0.8	30.5
terbutryn	V.18	180	3093	22.2	1.1	49.6
terbutryn	V.18	240	4035	20.6	1.4	46.1
terbutryn	V.18	315	5184	14.7	1.9	32.8
terbutryn	V.18	390	6296	18.6	2.2	41.6
terbutryn	V.18	495	7779	14.7	2.8	32.9
terbutryn	V.18	600	9187	8.9	3.3	20.0

Table S8. Mean concentrations and standard deviations (n = 3) of adsorption isotherms (data to Fig. 3).

Tracer	Site.Age	$c_{solution}$ ($\mu g L^{-1}$)	$q_{adsorbed}$ ($g kg^{-1}$)
UR	F.3	10 ± 0.6	49 ± 3.0
UR	F.3	13 ± 0.5	58 ± 2.4
UR	F.3	17 ± 0.2	67 ± 1.2
UR	F.3	20 ± 0.8	75 ± 4.0
UR	F.3	24 ± 2.0	82 ± 10.2
UR	F.3	25 ± 0.2	100 ± 1.0
UR	W.10	11 ± 0.3	46 ± 1.6
UR	W.10	13 ± 0.2	58 ± 1.0
UR	W.10	17 ± 0.4	66 ± 2.0
UR	W.10	22 ± 4.4	66 ± 22.1
UR	W.10	22 ± 0.7	88 ± 3.3
UR	W.10	24 ± 0.8	104 ± 4.0
UR	V.18	12 ± 1.6	38 ± 8.2
UR	V.18	13 ± 0.2	58 ± 1.2
UR	V.18	17 ± 0.6	67 ± 2.8
UR	V.18	20 ± 0.9	77 ± 4.5
UR	V.18	23 ± 0.8	87 ± 4.0
UR	V.18	23 ± 0.7	108 ± 3.7
SRB	F.3	144 ± 10.1	1281 ± 50.4
SRB	F.3	194 ± 7.4	1530 ± 36.9
SRB	F.3	234 ± 10.8	1828 ± 53.8
SRB	F.3	276 ± 0.9	2118 ± 4.3
SRB	F.3	329 ± 18	2356 ± 90.2
SRB	F.3	364 ± 7.3	2681 ± 36.7
SRB	W.10	129 ± 5.9	1356 ± 29.6
SRB	W.10	162 ± 7.5	1688 ± 37.7
SRB	W.10	197 ± 0.8	2014 ± 4.2
SRB	W.10	242 ± 4.3	2289 ± 21.3
SRB	W.10	274 ± 4.4	2631 ± 22.1
SRB	W.10	298 ± 13.7	3010 ± 68.3
SRB	V.18	128 ± 4.9	1358 ± 24.7
SRB	V.18	157 ± 3.9	1713 ± 19.6
SRB	V.18	194 ± 5.3	2029 ± 26.5
SRB	V.18	233 ± 13.6	2337 ± 68.2
SRB	V.18	258 ± 10.2	2709 ± 50.8
SRB	V.18	281 ± 8.2	3097 ± 41.1
